# Personality trait structures across three species of *Macaca*, using survey ratings of responses to conspecifics and humans

**DOI:** 10.1371/journal.pone.0309946

**Published:** 2024-09-06

**Authors:** Alexander J. Pritchard, Eliza Bliss-Moreau, Krishna N. Balasubramaniam, John P. Capitanio, Pascal R. Marty, Stefano S. K. Kaburu, Małgorzata E. Arlet, Brianne A. Beisner, Brenda McCowan

**Affiliations:** 1 California National Primate Research Center, University of California Davis, Davis, CA, United States of America; 2 Department of Population Health & Reproduction, School of Veterinary Medicine, University of California Davis, Davis, CA, United States of America; 3 Department of Psychology, University of California Davis, Davis, CA, United States of America; 4 School of Life Sciences, Faculty of Science & Engineering, Anglia Ruskin University, Cambridge, United Kingdom; 5 Nature Reserve and Wildlife Park Goldau, Goldau, Switzerland; 6 School of Animal, Rural and Environmental Sciences, Nottingham Trent University, Nottingham, United Kingdom; 7 Institute of Human Biology and Evolution, Faculty of Biology, Adam Mickiewicz University, Poznań, Poland; PLoS ONE, UNITED STATES OF AMERICA

## Abstract

Comparative studies reliant on single personality surveys to rate wild primates are scarce yet remain critical for developing a holistic comparative understanding of personality. Differences in survey design, item exclusion, and factor selection impede cross-study comparisons. To address these challenges, we used consistently collected data to assess personality trait structures in wild rhesus (*Macaca mulatta*), bonnet (*M*. *radiata*), and long-tailed (*M*. *fascicularis*) macaques that varied in their degree of phylogenetic closeness, species-typical social styles, and anthropogenic exposure in urban or urban-rural environments. We administered 51-item personality surveys to familiar raters, and, after reliability and structure screenings, isolated 4–5 factor solutions among the species. Four consistent factors emerged: Confident, Sociable, Active, and Irritable/Equable. This latter factor had differential expression across species. Item composition of the Irritable/Equable factor was consistent with their anticipated differences in social styles, but confounded by cross-site anthropogenic variation. We also administered a 43-item survey confined to human-primate situations which paralleled our findings of social style variation, while also exhibiting variation that aligned with population differences in human density. Our findings indicate that macaque personality trait structures may be emergent outcomes of evolutionary and/or socioecological processes, but further research is needed to parse these processes’ relative contributions.

## Introduction

Personality remains a salient and pervasive construct that can be helpful in understanding consistent individual differences in humans and other animals [1–8]. Despite the interest in nonhuman primate personality [4], 61% of studies prior to 2010 were only representative of rhesus macaques (*Macaca mulatta*) or chimpanzees (*Pan troglodytes*) [4]. After 2010, still only 52% of studies were represented by five species [7]. Additionally, the environmental condition of primate personality study subjects is predominantly captive, with wild subjects represented in only 9% of studies prior to 2010 [4], with some improvement to 25% since 2010 [7]. Addressing these gaps in phylogenetic diversity and environmental condition is important for understanding the evolution, biobehavioral expression, and function of personality. This deeper understanding of comparative personality could address questions about the processes of social evolution [6] (i.e., how does individual variation differ across species, and along which axes?), but is also expected to improve wild animal welfare and health outcomes, especially in environments where wildlife live near or frequently interact with humans [9–11] (i.e., are particular individuals, population, or species more susceptible to human impacts due to axes of individual variation or constraints?–a point we explore below).

For wildlife living in dynamic and challenging anthropogenic environments, inter-individual variation in, for example, sociodemographic characteristics or socio-spatial behavior has been shown to influence the relative propensity for risk-taking [12, 13]. Such wildlife living in synanthropy or sympatry with humans are often identified as ‘invasive’ and have been associated with high aggression, boldness, and activity [9, 14]. For example, heightened exploratory tendencies in Barbary ground squirrels (*Atlantoxerus getulus*) have been posited to be advantageous during island invasions, providing benefits in predator poor areas [15, 16]. Furthermore, such suites of traits have also been posited to generally increase in urban populations [17, 18]. In a social animal, individual tendencies can alter group- or population-level composition, and thereby influence which personality types succeed in a group [19–21]. These processes are complicated, however, in that individual animals and species show variation in the likelihood of persisting amidst, and taking risks during, heightened human proximity [12–14]. Thus, understanding the underlying personality structure across- and within-species is important from an applied animal behavior perspective; fundamental research on human-wildlife interactions would be advanced through a nuanced understanding of why individual animals, populations, or species are resilient to human encroachment, while others are more vulnerable.

Addressing these gaps, we explored personality structure *in situ* in ten wild groups of urban or urban-rural nonhuman primates, across three different species: rhesus macaques, bonnet macaques (*M*. *radiata*), and long-tailed macaques (*M*. *fascicularis*). Relative to captive populations, wild populations are important given evidence of greater variation in reactive physiology [22] and in the phenotypic expression of personality traits [23, 24]. We focused on free-living populations of these species within their natural ranges that also overlapped with humans and anthropogenic landscapes to varying extents. The genus *Macaca* was well suited for our objectives, due to its historically prolonged synanthropic associations with people at human-wildlife interfaces [25–27]. Furthermore, macaques have been shown to vary in social tolerance, which has historically led to researchers grouping them into ‘grades’ of social style [28, 29], although the latter is now understood to be more of a ‘continuum’ [30, 31]. At the interspecific level, these social styles have been shown to be broadly (albeit somewhat inconsistently) influenced by phylogenetic relatedness. For instance, degrees of social tolerance among female long-tailed macaques (that form the core of macaque groups) are expected to be relatively intermediate to the degrees of tolerance present among the more socially despotic rhesus macaques and the more tolerant bonnet macaques [28, 29]. This framework is complicated, however, by a less clear effect of phylogenetic co-variation in male-male tolerance, with evidence of the opposite pattern [32]. Bonnet macaques diverged from a shared ancestor 3.5 million years ago and so are more distantly related to long-tailed and rhesus macaques that together form a monophyletic group to which bonnet macaques are basal [33]. Finally, bonnet macaques have lower instances of interactions with humans (specifically human-directed aggression) in many parts of their range, compared to rhesus and long-tailed macaques [12, 34, 35].

Although our study is exploratory in nature, it is embedded within a robust body of supporting literature. Specifically, studies with an explicit comparative personality rating design have included: Assamese macaques (*M*. *assamensis*), Barbary macaques (*M*. *sylvanus*), Japanese macaques (*M*. *fuscata*), rhesus macaques, Sulawesi black crested macaques (*M*. *nigra*), and Tonkean macaques (*M*. *tonkeana*) [36–38]. Furthermore, we acknowledge a diversity of personality assessments on other macaques species [39–48].

Findings from comparative approaches can offer critical insights into shared and divergent personality structure. For instance, Adams et al. [36] analyzed ratings data from personality assessments on six macaque species. Their questionnaire was based off the human personality Five Factor Model [49–51], which is defined by the factors of Agreeableness, Conscientiousness, Extraversion, Openness, and Neuroticism. They found Friendliness (representing facets of Extraversion and Agreeableness) across all six species of *Macaca* and Openness across five species [36, 48]. They also found variation in personality structure such that species with a despotic social style clustered more closely together relative to the remaining social styles [36]. Similarly, Sussman et al. [47] reported that the despotic rhesus macaques exhibited greater scores on an aggressiveness component, and had aggressive behaviors contribute to all behaviorally coded components, relative to long-tailed macaques.

We also aimed to explore how human presence might mediate trait expression. We included this aim due to known variance in risk-taking behaviors attributed to anthropogenic effects [12–14]. While nonhuman personality ratings are posited to homogenize variation across contexts [4], there is a robust literature that suggests situations can alter the expression of personality in humans and animals [5, 52–54]. Indeed, individual differences in rhesus macaques’ Sociability interact with social housing situations to influence disease susceptibility [55, 56]. Variation of personality trait expression in response to distinct situations has been poorly described in the wild. Evidence from wild Tibetan macaques (*M*. *thibetana*), however, suggests that behaviors relevant to Insecurity, Boldness, and Sociability personality factors exhibit situational variance–contingent on the presence-absence of tourists and provisioning [42]. Similarly, comparisons between personality factor structures from two populations of Barbary macaques found evidence of an Opportunism factor in the provisioned population [40, 46]. Also in Barbary macaques, Baker et al. [37] identified a human-animal sociability factor that was associated with latency to touch a novel object. This factor was posited to be due to a reduced fear of items provisioned by human caregivers. Similarly, exposure trials to human testers found significant cross-species differences in a ‘Sociability towards Humans’ factor whereby long-tailed macaques exhibited negative scores, rhesus macaques were intermediate, and pig-tailed macaques (*M*. *nemestrina*) exhibited positive scores [47].

Due to the aforementioned evidence of such variance [37, 47], we included ratings on how individual macaques varied in response to humans. Human personality research has utilized assessments that draw upon experiences during specific situations [57, 58]. These assessments of specific situations provide a powerful tool to examine whether and which personality traits vary across situations. Thus, we sought to compare how personality factors from interactions with conspecifics, differed from personality factors in situations defined by interactions by macaques with humans.

We sought to accomplish three aims. Across three species of wild macaques living in anthropogenic urban or urban-rural environments, (1) we compared personality structure across the three species, we utilized the same rating system, facilitating quantitative and qualitative comparisons. The use of the same methodology has been emphasized as a priority in comparative personality work on wild animals [46]. (2) We compared situation-specific differences in personality structure. To this end, we developed personality ratings constrained to situations involving interactions between humans and our subjects. (3) Finally, we compared individual scores in the resulting factor models from aim 1 and aim 2. Although this comparative study was largely exploratory, we predicted that personality structure would exhibit greater structural similarity across species consistent with their relative similarities in species-typical social styles and phylogenetic relatedness. That is, long-tailed macaques would structurally fall between the more phylogenetically similar rhesus macaques and the more phylogenetically basal bonnet macaques–such intermediate placement would be attributable to their phylogenetic relatedness, but also their relative social style. Similarly, we expected that species which occupied more urbanized areas (i.e., long-tailed and rhesus macaques, relative to bonnet macaques) would exhibit personality structures more strongly defined by aggressive, bold, and active traits [9, 14].

## Methods

### Site and study groups

We studied adult and sub-adult monkeys from three species at three different sites: rhesus macaques at Shimla, India (31.05° N, 77.1° E), long-tailed macaques at Batu Caves and Templer Park, Malaysia (3.23° N, 101.7° E and 3.29° N, 101.6° E, respectively), and bonnet macaques near Thenmala, India (8.90° N, 77.10° E). The bonnet macaques were observed nearby to a small town (Thenmala) and had reduced access to anthropogenic food compared to the long-tailed and rhesus macaques that occupied sites near cities (Kuala Lumpur and Shimla, respectively) [59]. The parks that the long-tailed macaques occupied, despite geographic proximity, had marked differences in the number of tourist visitors per day [60]. Behavioral data collection was conducted between 2016–2018. We obtained ratings from a total of 328 wild subjects. Group and sex breakdowns are available in **Table 1** with further site and subject details available elsewhere [12, 59, 61, 62].

**Table 1:** Adult study subjects, by group and sex.

Social Group	Rated Subjects (N)		
	Females	Males	Total
	**Rhesus macaques**		
** Hook**	17	9	26
** Mall**	16	8	24
** Ripped-Ear**	28	13	41
** Shaggy**	44	15	59
**Total**	105	45	150
	**Long-tailed macaques**		
** Entrance**	19	8	27
** Hulk**	12	7	19
** Lip**	19	13	32
** Pirate**	18	5	23
**Total**	68	33	101
	**Bonnet macaques**		
** Dam**	23	27	50
** Eco**	18	9	27
**Total**	41	36	77

### Survey

We used a personality survey previously published in Maninger et al. [63]. Their survey was assembled from published nonhuman personality studies available at the time [44, 50, 64, 65] in addition to item contributions from animal husbandry technicians. These surveys were originally constructed primarily via a behavioral bottom-up approach for items (i.e., derived from adjectives and definitions that describe observable behavior of the relevant species) [66]; definitions were constructed or modified with careful consideration of the study species and circumstances. The Maninger et al.’s [63] survey included 50 items. We modified the surveys as follows. First, we omitted 5 items: cooperative, flexible/not rigid, helpful, imaginative/creative, and imitative. Then, because the present study included females, we added 6 items and their associated definitions from Stevenson-Hinde et al. [44]. These items were apprehensive, effective, equable, sensitive, sociable, and strong. Finally, we added the item feisty, with a descriptor of: “having or showing lively aggressiveness; spunky.”

Sites differed in the number of observers who contributed: five observers rated rhesus macaques, four rated long-tailed macaques, and five rated the bonnet macaques. Raters were specific to each site and, within each site, not every observer rated every subject. Raters were extensively trained on the survey tool by EBM to ensure they understood the meaning of each adjective, could think of examples of behaviors related to low/average/high ratings of each adjective (without referencing specific animals familiar to observers), and understood the difference between ratings being made of the animals in general versus the animals in human context. Scenarios were presented, without referencing specific animals, to aid in defining what would constitute high and low scores. This process required back-and-forth translation between English (the language of the ratings) and the languages that were spoken by the members of the data collection teams (all raters were fluent in English). EBM engaged in an iterative process at each site to ensure everyone was trained in the same way and understood the protocols. Subjects were the same between the general and human situation ratings, though one long-tailed macaque subject was missing a rating for sociable in the human situation.

All field observers who completed the surveys were familiar with all individual animals; they collected data on activity budgets and social behavior using focal animal sampling of individual animals [67] for 18–20 months in the rhesus and long-tailed macaques and 11 months in the bonnet macaques [12, 59, 61, 62]. During these data collection periods, observers were asked to rate each subject on adjectives with associated descriptions, and to limit discussion about the rating of specific monkeys. Raters were asked to skip subjects that they were not confident that they knew. Ratings were collected after all behavioral data were collected and not at the locale at which behavioral data were collected; ratings were based on subjects in general, rather than particular instances of behavior. Eight items were excluded from the human situation ratings: jealous, manipulative, nurturant, playful, popular, protective, stingy/greedy, and warm/affectionate. These adjectives were excluded during the training process prior to scoring individual animals after the team was unable to generate specific examples of behavior relating to the adjectives. Observers completed the general and human situation ratings in parallel.

Despite the extensive training prior to ratings, at one of the sites EBM observed conversation between raters during their completion of human situation ratings (but not the general ratings). This observation suggests that ratings that sample were potentially not independent, which we inferred to be due to a greater difficulty in completing human situation ratings. Personal communications (December 8, 2023) with multiple raters from other sites suggests that this was not a uniform occurrence. Thus, we have retained these data.

### Data processing and analyses

All data processing and analyses were conducted using R (v4.2.2) [68]. We assessed inter-rater reliability for each of the items using intraclass correlations via the ICC() function in the *psych* package [69]. As our aim was to aggregate ratings across raters, we relied on ICC(3,k) following guidelines and underlying rationale detailed elsewhere [70–74]. We also report ICC(3,1). The two estimates are mathematically similar [71, 75], though their interpretation differs [70, 75]. ICC(3,k) assesses the reliability of a composite for use with mean ratings [71, 73, 74], while ICC(3,1) is important for individual rater reliabilities [71, 73]. We dropped items that had an *a priori* cut-off of ICC(3,k) ≤ 0.40. A cut-off of 0.40 follows published recommendations for scales [76]. Though single items are expected to have lower reliabilities than scales [77], ICC(3,k) has higher estimates than ICC(3,1) [78] with measurement error reduced through the process of averaging ratings [78]. Generally, however, ICC cut-offs range across studies with researchers identifying liberal cut-offs of ICC > 0.00, while others are more conservative (i.e., ICC[3,k] > 0.50 or > 0.70) [e.g., 72, 77, 79, 80]. We replicated our analyses to verify that our reliability threshold or use of ICC(3,k) was not overly informing our model outcomes.

We conducted factor analyses independently for each of the three species using *fa()* function within the *psych* package [69]. The process was identical for each dataset. We chose to analyze each species separately rather than pool the datasets, primarily because we had no *a priori* rationale to assume that factor structure would be identical across the three species.

For each factor model, we first averaged raters’ scores across each subject, so that every monkey only had a single rating for each item. We utilized a similar model selection procedure across species to facilitate comparability. We followed recommendations for assessing the number of factors to extract and model fit [69, 81, 82]. First, we extracted Kaiser, Meyer, Olkin Measures of Sampling Adequacy (MSA) for each item and discarded items < 0.50. We then used parallel analysis, empirical BIC, and very simple structure (complexity of 1) to interrogate the probable number of factors. If there was a discrepancy between these estimates, we took the median number of factors. We removed any items whose communality–a measure of the proportion of item variance explained by the factor model–was ≤ 0.4 or ≥ 0.99. We then confirmed each item had an MSA ≥ 0.50 and that the resulting models’ square root of the sum of squared correlation residuals was < 0.10. We relied on minimum residuals solutions and used oblique (oblimin) transformations throughout. We ran this process for each of the three species’ general and human situation ratings, resulting in six factor models. We selected factor names from heavily loading items within each factor, with some consideration of whether there were comparable factors in the other species.

#### Comparing factor models

Raters were asked to mean-center their ratings; thus, even without scaling, comparisons across datasets are not as straightforward as analyzing average ratings across the models. Therefore, we relied on comparisons of structural equivalence through two methods of comparison: 1) obtaining Pearson’s correlation and Tucker’s congruence coefficients from the factor loadings for items present in species’ factor models, 2) calculating fuzzy set intersections for all the items present in any of the species’ factor models. The former approach provides insight as to whether factors show quantitatively similar loadings, and is conducted pairwise between the species. The fuzzy set analysis, instead, assumes a similar factor structure across all species providing insight into the intersection of composite factor items. That is, these fuzzy set analyses are important for examining which items are likely to be representative of these factors across all three species, rather than defining factor structure within a species.

To obtain correlation and congruence coefficients, we followed recommendations [83] to performed pairwise Procrustes rotations (using the *Procrustes()* function [69]) between the species using their loading matrices. Loading matrices were restricted to items present in both species’ models. We then calculated congruence coefficients using the *fa*.*congruence()* function and correlation coefficients using the *cor()* function in the psych [69] and stats [68] R packages. We recognize standards of a consensus-derived cut-off of 0.85 for congruent versus incongruent set within a single study [83], but that 0.80 has been posited to be an agreeable cut-off across studies [84]. We are foremost focused on similarity, rather than absolute congruence–even so, we use *fair* 0.85–0.94 and *good* congruence (0.95–1.00), and a hard cut-off for congruence of 0.80. A score of 0.80 was intermediate between *fair* and *poor* [83]. For transparency, we use *poor* congruence, following the five point scale bins used by Lorenzo-Seva and ten Berge [83], but note that their point estimate of *poor* was approximated by 0.72. The use of 0.80 is not without precedent [84] in, for example, cross-cultural work [85]. This caveat is relevant here because we assessed subjects across species boundaries and raters themselves were cross-cultural.

Fuzzy set intersections [86, 87] were calculated using R code provided in Adams et al. [36]. Thus, we examined which items intersected with factors that were structurally similar across species. For example, if we found a dimension such as Extraversion across three species, we might be interested in which items showed the greatest support across the three groups for heavily contributing to Extraversion. Intersections were functionally equivalent to the minimum absolute loading for valid items in any given factor’s fuzzy set. Thus, in the prior example, if species’ loadings for the item sociable within the factor Extraversion were 0.12, 0.25, 0.76, then the fuzzy intersection for sociable would be 0.12 for Extraversion. This provides a conservative estimate of concordance for items that compose factors across multiple species. That is, fuzzy intersections represent the minimum estimate of mutually shared space. For items that are not present due to lack of reliability in one or more species, the estimate is drawn from known information (i.e., the loadings from species that had the items as reliable). Before running fuzzy set analyses, we reverse scored item loadings such that factors were directionally consistent across species.

As fuzzy intersection scores are drawn from the loadings themselves, they range from -1 to 1 per item, and can be interpreted similarly to factor loadings [36]. We followed a similar approach to that reported elsewhere to determining which items were salient for a fuzzy set [36]. We conducted 1000 permutations, where we selected a random factor from each species for each iteration. We extracted all the fuzzy item scores for each randomly extracted fuzzy set. We then took the 95^th^ percentile of the absolute value of all permuted iterations. We conducted the same procedure within the personality factor models and the human factor models and then aggregated the fuzzy item scores. The 95^th^ percentile for these scores was 0.43. To confirm neither the personality factor models nor the human situation models were overly influential towards this cut-off, we calculated the 95^th^ percentile within each (0.36 and 0.49, respectively). This cut-off was not overly dissimilar from 0.40, which is frequently used as meaningful for factor loadings. Thus, we simplified our judgement for salient fuzzy set scores to a threshold of ≥|0.40|.

Finally, though the average item ratings showed high association between the two survey results, we recognize that factor analyses may result in the emergence of latent structures that are distinct between the two datasets. Thus, we compared subjects’ factors scores from the general factors to those from the human situation ratings. To compare the factor structures from the general (conspecific) personality ratings and the human situation personality ratings, we computed factor scores for each subject using ten Berge’s estimation [88]. Then, for each of the taxa, we computed Pearson’s correlation coefficients across the factor scores in the general versus human situations. As previously noted, one long-tailed macaque subject was excluded from these comparisons due to a missing rating in the human situation.

## Results

### General conspecific personality ratings

The following items fell below our *a priori* interrater reliability cut-off and were omitted: reckless, sensitive, unemotional, and vigilant for rhesus macaques; excitable, sensitive, and unpredictable for long-tailed macaques; and fearful, independent, insecure, sensitive, tolerant, and vigilant for bonnet macaques (**Table 2; S1 Table in S1 File**). The mean average for retained items was 0.68 ±0.12*sd* for ICC(3,k) and 0.34 ±0.14*sd* for ICC(3,1) (**S2 Table in S1 File**). Replicated analyses with varying ICC cut-offs verified that our reliability threshold did not overly influence our model outcomes (**S1 Text; S1 Fig; S3 Table in S1 File**).

**Table 2:** Results from three factor analyses on the general ratings, with a separate factor model from each species (S5-S7 Tables in S1 File). Criteria of item exclusion are annotated. Factors ordered by rhesus macaque structure.

	Rhesus macaques					Long-tailed macaques					Bonnet macaques			
Items	Irritable^R^	Confident^R^	Sociable^R^	Active^R^	Equable^R^	Confident^L^	Sociable^L^	Irritable/ Equable^L^	Active^L^	Playful^L^	Confident^B^	Active^B^	Sociable^B^	Equable^B^
Irritable	**0.84**	-0.01	-0.06	-0.02	-0.22	-0.04	0.03	**0.95**	-0.12	0.04	**0.41**	0.00	-0.06	**0.66**
Bullying	**0.79**	-0.19	0.03	-0.06	-0.08	0.36	0.05	**0.66**	0.10	0.24	**0.81**	-0.13	0.01	**0.45**
Excitable	**0.77**	0.08	0.04	-0.15	-0.09	Low ICC					-0.16	**0.70**	0.04	**0.47**
Jealous	**0.77**	-0.13	0.18	0.00	-0.12	0.33	0.22	**0.54**	0.06	0.32	**0.66**	-0.06	0.27	**0.44**
Stingy/greedy	**0.75**	-0.13	0.05	0.14	-0.11	**0.58**	-0.12	0.46	0.10	0.22	**0.58**	-0.12	0.16	0.18
Feisty	**0.73**	-0.06	0.11	-0.18	-0.15	0.12	0.03	**0.84**	0.09	0.08	**0.64**	0.19	-0.16	**0.49**
Unpredictable	**0.70**	0.31	-0.19	-0.27	0.12	Low ICC					0.21	**0.45**	**-0.49**	0.33
Aggressive	**0.67**	-0.30	-0.01	-0.05	-0.17	**0.40**	0.10	**0.70**	0.07	0.23	**0.87**	-0.13	-0.06	0.34
Persistent	**0.64**	-0.21	0.15	-0.02	0.04	0.24	0.13	0.26	**0.48**	0.35	**0.77**	0.23	0.02	0.12
Defiant	**0.62**	0.00	0.22	-0.17	0.01	0.14	0.15	0.37	0.38	0.21	**0.80**	0.19	0.13	0.08
Impulsive	**0.54**	0.18	-0.19	**-0.47**	0.27	-0.05	-0.06	**0.43**	**0.55**	0.13	0.25	**0.57**	-0.31	**0.40**
Opportunistic	**0.44**	-0.13	0.19	-0.23	0.20	0.08	0.12	0.12	**0.64**	0.26	0.36	**0.43**	0.27	0.23
Fearful	0.03	**0.91**	-0.02	0.09	0.06	**-0.83**	-0.12	0.08	-0.15	0.02	Low ICC			
Apprehensive	0.08	**0.91**	0.11	0.10	0.02	**-0.89**	-0.05	0.04	-0.14	0.13	**-0.52**	-0.23	-0.35	0.37
Nervous/anxious/not calm	0.34	**0.85**	0.04	-0.06	0.02	**-0.74**	-0.05	0.31	0.05	0.10	**-0.51**	0.06	-0.22	**0.62**
Insecure	0.12	**0.75**	0.37	0.10	-0.12	**-0.75**	**0.44**	0.21	-0.06	-0.04	Low ICC			
Submissive/subordinate	-0.14	**0.70**	-0.22	0.11	0.27	**-0.96**	-0.06	-0.05	0.15	0.01	**-0.87**	0.16	-0.15	0.10
Timid	-0.14	**0.69**	-0.03	0.26	0.17	**-0.83**	-0.05	-0.01	-0.15	0.07	**-0.73**	-0.23	-0.20	0.23
Cautious	-0.13	**0.68**	-0.09	0.20	0.18	**-0.81**	-0.02	0.00	-0.20	0.04	**-0.78**	-0.38	0.04	0.12
Protective	0.21	**-0.53**	0.38	0.15	0.24	0.20	**0.75**	0.17	-0.04	0.20	**0.77**	-0.22	0.26	-0.09
Intelligent	0.09	**-0.54**	0.18	-0.25	**0.43**	Low Communality					**0.59**	-0.01	0.24	-0.31
Bold	**0.54**	**-0.58**	0.06	0.00	0.11	**0.82**	0.03	0.11	0.06	0.18	**0.74**	**0.42**	-0.02	-0.11
Direct/forceful/gets own way	**0.48**	**-0.63**	0.15	0.06	0.04	**0.81**	0.11	0.19	-0.02	0.21	**0.92**	-0.10	0.07	-0.01
Confident	**0.40**	**-0.66**	0.13	-0.02	0.10	**0.90**	0.06	-0.01	0.01	0.11	**0.77**	0.26	0.18	-0.21
Strong	0.29	**-0.68**	-0.22	0.14	0.33	**0.54**	-0.04	0.14	0.10	**0.46**	**0.87**	-0.06	**-0.44**	-0.15
Effective	0.36	**-0.72**	0.19	0.13	0.03	**0.78**	0.18	0.12	0.08	0.16	**0.95**	-0.10	0.03	0.03
Sociable	0.18	0.00	**0.89**	0.06	0.07	0.18	**0.87**	-0.03	0.08	-0.01	0.24	0.23	**0.76**	0.03
Affiliative/Companionable	0.14	0.10	**0.88**	0.09	0.15	0.20	**0.82**	-0.09	0.11	0.03	0.19	0.20	**0.80**	0.03
Warm/affectionate	0.01	0.13	**0.79**	0.01	0.36	0.02	**0.91**	-0.10	0.00	0.06	0.01	-0.10	**0.81**	-0.13
Nurturant	-0.08	0.07	**0.71**	0.03	0.35	-0.18	**0.91**	-0.09	-0.03	0.11	-0.09	-0.18	**0.80**	-0.10
Popular	0.11	-0.38	**0.68**	-0.08	0.11	**0.46**	**0.67**	-0.03	-0.04	0.11	**0.58**	0.14	**0.50**	-0.02
Manipulative	**0.48**	-0.01	**0.54**	-0.05	-0.17	0.11	0.31	0.31	0.25	0.34	**0.72**	-0.10	0.16	0.23
Independent	0.28	**-0.43**	**-0.47**	-0.05	0.39	0.31	**-0.80**	-0.15	0.00	**0.41**	Low ICC			
Depressed	0.12	0.26	**-0.56**	**0.49**	0.24	**-0.62**	-0.30	0.08	-0.32	0.30	-0.20	**-0.55**	**-0.52**	0.01
Solitary	0.17	0.14	**-0.72**	0.13	**0.45**	**-0.43**	**-0.75**	-0.04	-0.04	0.26	-0.15	-0.15	**-0.81**	-0.14
Lazy	0.06	0.10	-0.04	**0.90**	0.11	-0.22	-0.10	0.01	**-0.75**	0.31	-0.01	**-0.78**	-0.34	-0.08
Slow	-0.01	0.04	-0.04	**0.86**	0.16	0.17	0.13	-0.09	**-0.79**	0.29	0.06	**-0.80**	-0.30	-0.14
Curious/exploratory/inquisitive	0.04	0.00	0.01	**-0.63**	**0.50**	0.08	0.05	-0.10	**0.70**	0.29	0.18	**0.87**	-0.04	-0.16
Active/Energetic	0.25	-0.05	-0.18	**-0.78**	0.18	0.03	-0.09	-0.03	**0.88**	0.10	0.00	**0.85**	0.15	0.06
Tolerant	-0.30	0.10	0.30	0.11	**0.65**	-0.10	**0.59**	**-0.54**	0.08	0.25	Low ICC			
Equable	-0.34	-0.08	0.13	0.22	**0.65**	0.29	0.15	**-0.76**	-0.16	0.22	0.20	-0.08	0.04	**-0.81**
Understanding	-0.19	-0.21	0.35	-0.03	**0.65**	0.13	0.23	**-0.64**	0.09	0.03	0.03	-0.24	**0.59**	-0.35
Calm/Equable	-0.33	-0.09	0.13	0.35	**0.61**	0.27	0.14	**-0.8**	-0.15	0.17	-0.02	-0.25	0.06	**-0.68**
Playful	-0.18	0.10	0.11	**-0.42**	**0.58**	-0.15	0.24	-0.16	0.39	**0.53**	-0.30	**0.95**	-0.12	-0.14
Gentle	**-0.41**	0.12	0.32	0.15	**0.57**	-0.28	**0.50**	**-0.57**	-0.06	0.25	**-0.45**	0.09	0.04	**-0.65**
Reckless	Low ICC					-0.01	-0.02	0.25	**0.54**	0.21	**0.44**	**0.62**	-0.34	0.19
Tense	Low Communality					-0.27	-0.13	**0.40**	**-0.55**	0.10	**-0.62**	-0.26	-0.19	0.36
Unemotional	Low ICC					**0.40**	-0.10	**-0.53**	-0.26	0.26	-0.10	-0.34	**-0.47**	**-0.40**
Eccentric	Low Communality					Low Communality					0.12	-0.06	**-0.63**	0.13
Sensitive	Low ICC					Low ICC					Low ICC			
Vigilant	Low ICC					Low Communality					Low ICC			

Several items were excluded due to low communality. For the rhesus macaques, we excluded eccentric and tense due to low communality. For the long-tailed macaques, we excluded eccentric, intelligent, and vigilant due to low communality. Finally, for the bonnet macaques, no additional items were excluded.

From the original 51 items, we retained 45 for five factor solutions in the long-tailed and rhesus macaque models, and 45 for a four-factor solution in the bonnet macaque model. For the factor names, we have appended a superscript for each of the relevant species (‘R’ for rhesus macaques, ‘L’ for long-tailed, and ‘B’ for bonnet). The rhesus macaque dataset supported a five-factor solution (**Table 2; S5 Table in S1 File**), with the factors explaining 73% of the cumulative variation in the data. These factors were named as follows, with the proportion of variation explained in parentheses: Irritable^R^ (0.20); Confident^R^ (0.20); Sociable^R^ (0.13); Active^R^ (0.10); and Equable^R^ (0.10). The long-tailed macaque dataset supported a five-factor solution (**Table 2; S6 Table in S1 File**), with the factors explaining 76% of the cumulative variation in the data. Factor names and the proportion of variation explained were: Confident^L^ (0.26); Sociable^L^ (0.16); Irritable/Equable^L^ (0.16); Active^L^ (0.12); and Playful^L^ (0.06). The bonnet macaque dataset supported a four-factor solution (**Table 2; S7 Table in S1 File**), with the factors explaining 75% of the cumulative variation in the data. Factor names and the proportion of variation explained were: Confident^B^ (0.32); Active^B^ (0.16); Sociable^B^ (0.16); and Equable^B^ (0.11).

The models had low root mean square residuals (RMSR) with a degrees of freedom correction (0.04, 0.03, and 0.03 for the bonnet, long-tailed, and rhesus macaque models, respectively), and were all below 0.08 indicating acceptable goodness-of-fit. Alpha and total omega estimates were high across the three models (≥ 0.95). Communalities were high, with means of 0.73 ±0.11*sd* for rhesus, 0.76 ±0.13*sd* for long-tailed, and 0.75 ±0.12*sd* for bonnet macaques. We present interfactor correlation coefficients in the supplementary (**S8 Table in S1 File**). Our model structures relative to item and subject sample sizes were sufficient based on screening recommendations for communality values, item:factor ratios, and sample size [89].

The final models retained several complex items (i.e., items with moderate-to-heavy loadings across multiple factors). Construction of simple models with sequential omission of complex items were highly similar in their factor loadings and resulting structure (**S2 Text; S9 Table in S1 File**). Additionally, lower reliability cut-offs (i.e., ICC[3,1] ≤ 0.10 or ≤ 0.00) did not alter our model structures (**S1 Text; S1 Fig; S3 Table in S1 File**). Thus, we proceeded with full models containing complex items to prioritize item retention and comparability between the structures.

#### Species comparisons of general conspecific factor models

To compare structures, we first merged factor loading matrices by pairs for all three of the factor models and conducted cross-correlations on items present in all models (**Table 3**). We observed high cross-correlations in Active^RBL^, Confident^RBL^, and Sociable^RBL^ (whereby superscripts signify the species for which each factor is present with ‘RBL’ indicating rhesus, bonnet, and long-tailed macaques) evidencing structural comparability across the taxa, with an average |*r*| of 0.90. We also found high comparability for two factors, Equable and Irritable, with an average |*r*| between factor loadings of 0.86.

**Table 3:** For the personality models, Tucker’s congruence (Φ) and Pearson’s correlation coefficients (*r*) present in three matrices, each representative of pairwise cross-species comparisons between Procrustes rotated factor loadings.

	Φ	*r*	Φ	*r*	Φ	*r*	Φ	*r*	Φ	*r*
**Long-tailed by Rhesus macaques**										
	Irritable^R^		Confident^R^		Sociable^R^		Active^R^		Equable^R^	
Confident^L^	-0.26	-0.33	0.94	0.94	-0.15	-0.17	0.10	0.10	-0.01	-0.03
Sociable^L^	0.06	-0.15	-0.16	-0.18	0.94	0.93	-0.02	-0.06	0.21	0.02
Irritable/Equable^L^	0.95	0.94	-0.31	-0.36	0.07	-0.10	-0.24	-0.33	-0.26	-0.74
Active^L^	-0.23	-0.29	0.11	0.11	-0.02	-0.03	0.92	0.92	0.01	-0.01
Playful^L^	-0.28	-0.75	-0.01	-0.04	0.26	0.10	0.01	-0.06	0.90	0.87
**Long-tailed by Bonnet macaques**										
	Confident^B^		Active^B^		Sociable^B^		Equable^B^			
Confident^L^	0.88	0.90	0.23	0.22	0.36	0.35	0.25	0.23		
Sociable^L^	0.33	0.23	0.16	0.13	0.82	0.85	-0.02	-0.14		
Irritable/Equable^L^	0.23	0.17	0.18	0.17	-0.02	-0.04	0.94	0.94		
Active^L^	0.25	0.22	0.89	0.88	0.19	0.17	0.22	0.19		
Playful^L^	0.45	0.32	0.11	0.04	-0.12	-0.40	0.25	0.03		
**Rhesus by Bonnet macaques**										
	Confident^B^		Active^B^		Sociable^B^		Equable^B^			
Irritable^R^	-0.42	-0.28	-0.23	-0.20	0.11	0.00	0.91	0.92		
Confident^R^	0.89	0.93	0.02	0.01	-0.35	-0.35	-0.47	-0.49		
Sociable^R^	-0.33	-0.23	-0.12	-0.09	0.88	0.89	0.11	-0.03		
Active^R^	0.02	0.04	0.86	0.87	-0.14	-0.15	-0.28	-0.32		
Equable^R^	-0.18	0.03	-0.11	-0.05	0.06	-0.06	-0.31	-0.74		

We found a general pattern of *fair* congruence across Active^RBL^, Confident^RBL^, and Sociable^RBL^, with the exception of *poor* congruence for Sociable^BL^ (**Table 3**). Equable and Irritable exhibited more complex pairwise dynamics between the three species. We found *good* congruence between Irritable^R^ and Irritable/Equable^L^, as well as *fair* congruence between Equable^R^ and Playful^L^. Both Irritable^R^ and Irritable/Equable^L^ had *fair* congruence with Equable^B^, Both Equable^R^ and Playful^L^ were incongruent with all the bonnet macaque factors.

We also completed fuzzy set analyses on five factors that were consistent across the three species (Active^RBL^, Confident^RBL^, Sociable^RBL^, and Irritable^RL^ and/or Equable^RBL^) (**Table 4** and **S10 Table in S1 File**). As the item composition and presence of Irritable and Equable varied across species, we treated these as distinct factors with the understanding that the bonnet macaque factor structure was more heavily represented by Equable-like items, the rhesus macaque model had two distinct factors (Irritable and Equable), and the long-tailed macaque factor was composed of items from both factors (Irritable/Equable). We found that each of the five fuzzy sets had four or more items with values ≥ |0.40| and, therefore, were likely representative of major factors across species. We appended a superscript ‘F’ to distinguish fuzzy sets from factors. Using a threshold of ≥ |0.40|: Confident^F^ included apprehensive, bold, cautious, confident, direct/forceful/gets own way, effective, fearful, insecure, intelligent, nervous/anxious/not calm, strong, submissive/subordinate, and timid. Sociable^F^ included affiliative/companionable, eccentric, independent, nurturant, popular, sociable, solitary, and warm/affectionate. Active^F^ included active/energetic, curious/exploratory/inquisitive, impulsive, lazy, reckless, and slow. Irritable^F^ included bullying, excitable, feisty, irritable, and jealous. Equable^F^ included calm/equable, equable, gentle, and tolerant. We found evidence for similar item placement in each of these five factors, though we underscore the lack of distinct factors between Irritable and Equable across all three species.

**Table 4:** Items (in bold font) with scores ≥ |0.40| in our fuzzy set analyses for the four factors that appeared across all three species in our general factor models. Items are represented in all species models, unless otherwise indicated with superscript of the species where items are in the model (annotated with ^BLR^ for bonnet, long-tailed, or rhesus macaques, respectively). See **S10 Table in S1 File** for the remaining items.

Items	Fuzzy Sets				
	Confident^F^	Sociable^F^	Active^F^	Irritable^F^	Equable^F^
Effective	**0.72**	0.03	0.08	0.03	-0.03
Confident	**0.66**	0.06	0.01	-0.01	-0.01
Direct/forceful/ gets own way	**0.63**	0.07	-0.02	-0.01	-0.01
Bold	**0.58**	-0.02	0.00	0.11	0.11
Intelligent^BR^	**0.54**	0.18	-0.01	0.09	-0.31
Strong	**0.54**	-0.04	-0.06	0.14	0.14
Nervous/anxious/not calm	**-0.51**	0.04	0.05	0.31	-0.02
Apprehensive	**-0.52**	-0.05	-0.10	0.04	-0.02
Cautious	**-0.68**	-0.02	-0.20	0.00	0.00
Timid	**-0.69**	-0.03	-0.15	-0.01	-0.01
Submissive/subordinate	**-0.70**	-0.06	-0.11	-0.05	-0.05
Insecure^LR^	**-0.75**	0.37	-0.06	0.12	0.12
Fearful^LR^	**-0.83**	-0.02	-0.09	0.03	-0.06
Affiliative/Companionable	-0.10	**0.80**	-0.09	0.03	0.03
Warm/affectionate	0.01	**0.79**	0.00	0.01	-0.10
Sociable	0.00	**0.76**	-0.06	-0.03	-0.03
Nurturant	-0.07	**0.71**	-0.03	-0.08	-0.09
Popular	0.38	**0.50**	-0.04	-0.02	-0.02
Independent^LR^	0.31	**-0.47**	0.00	-0.15	-0.15
Eccentric^B^	0.12	**-0.63**	-0.06	0.13	0.13
Solitary	-0.14	**-0.72**	-0.04	-0.04	-0.04
Active/Energetic	0.00	-0.09	**0.78**	-0.03	-0.03
Curious/exploratory/inquisitive	0.00	0.01	**0.63**	0.04	-0.10
Reckless^BL^	-0.01	-0.02	**0.54**	0.19	0.19
Impulsive	-0.05	-0.06	**0.47**	**0.40**	-0.27
Lazy	-0.01	-0.04	**-0.75**	0.01	0.01
Slow	-0.04	-0.04	**-0.79**	-0.01	-0.09
Irritable	0.01	0.03	0.00	**0.66**	0.22
Feisty	0.06	0.03	0.09	**0.49**	0.15
Excitable^BR^	-0.08	0.04	0.15	**0.47**	0.09
Bullying	0.19	0.01	0.06	**0.45**	0.08
Jealous	0.13	0.18	0.00	**0.44**	0.12
Tolerant^LR^	-0.10	0.30	0.08	-0.30	**-0.54**
Gentle	-0.12	0.04	-0.06	**-0.41**	**-0.57**
Calm/Equable	-0.02	0.06	-0.15	-0.33	**-0.61**
Equable	0.08	0.04	-0.08	-0.34	**-0.65**

### Human situation personality ratings

The following items fell below our *a priori* reliability cut-off and were omitted: affiliative/companionable, sensitive, submissive/subordinate, and tense for rhesus macaques; affiliative/companionable, aggressive, bold, calm/equable, eccentric, equable, excitable, feisty, gentle, impulsive, independent, insecure, intelligent, irritable, sensitive, sociable, solitary, strong, tolerant, unemotional, unpredictable, and vigilant for bonnet macaques (**Table 5; S11 Table in S1 File**). For long-tailed macaques, none of the remaining items were excluded based on ICC(3,k). The mean average for reliable items was 0.63 ±0.12*sd* for ICC(3,k) and 0.29 ±0.12*sd* for ICC(3,1) (**S12 Table in S1 File**). Replicated analyses with varying ICC cut-offs verified that our reliability threshold did not overly influence our model outcomes (**S1 Text; S1 Fig; S4 Table in S1 File**).

**Table 5:** Results from three factor analyses on the human situation ratings, one on each species (S13-S15 Tables in S1 File). Criteria of item exclusion are annotated. Factors ordered by rhesus macaque structure.

	Rhesus macaques				Long-tailed macaques				Bonnet macaques		
Items	Irritable^R^_H_	Exploratory^R^_H_	Apprehensive^R^_H_	Lazy^R^_H_	Apprehensive^L^_H_	Exploratory^L^_H_	Irritable^L^_H_	Lazy^L^_H_	Effective^B^_H_	Lazy/ Exploratory^B^_H_	Apprehensive^B^_H_
Irritable	**0.92**	-0.02	0.00	0.27	0.03	-0.04	**0.89**	0.08	Low ICC		
Aggressive	**0.82**	0.11	0.08	0.17	-0.27	0.09	**0.84**	0.14	Low ICC		
Excitable	**0.81**	0.20	-0.01	0.18	0.20	0.28	**0.66**	-0.30	Low ICC		
Feisty	**0.80**	0.24	0.05	0.17	-0.22	0.02	**0.84**	0.05	Low ICC		
Bullying	**0.69**	0.32	0.21	0.13	-0.38	0.31	**0.58**	0.12	**0.78**	0.15	-0.04
Reckless	**0.65**	0.26	0.25	0.25	-0.10	**0.52**	**0.46**	-0.07	**0.63**	-0.36	0.28
Defiant	**0.45**	0.26	0.37	0.27	-0.19	**0.54**	**0.48**	0.01	**0.79**	-0.20	0.01
Unemotional	**-0.68**	0.13	-0.13	0.29	**-0.44**	0.01	**-0.51**	0.36	Low ICC		
Understanding	**-0.75**	**0.43**	0.10	0.11	-0.24	0.10	**-0.67**	-0.11	Low Communality		
Tolerant	**-0.82**	0.19	0.16	0.25	-0.12	**0.69**	**-0.55**	0.17	Low ICC		
Gentle	**-0.83**	-0.05	-0.01	0.27	0.27	**0.47**	**-0.59**	0.19	Low ICC		
Calm/Equable	**-0.86**	-0.02	0.07	0.29	-0.25	0.18	**-0.76**	0.23	Low ICC		
Equable	**-0.93**	0.18	0.06	0.20	-0.23	0.23	**-0.69**	0.32	Low ICC		
Curious/exploratory/inquisitive	-0.17	**0.86**	0.10	-0.09	-0.16	**0.78**	-0.01	-0.17	0.03	**-0.87**	-0.03
Vigilant	0.05	**0.84**	-0.20	-0.01	-0.13	0.37	0.18	**-0.47**	Low ICC		
Intelligent	-0.37	**0.80**	0.23	-0.05	**-0.41**	0.34	-0.08	-0.32	Low ICC		
Opportunistic	-0.04	**0.80**	0.17	0.03	-0.25	**0.65**	0.06	-0.15	0.28	**-0.56**	0.05
Active/Energetic	0.12	**0.76**	0.18	-0.21	-0.08	**0.72**	0.08	-0.27	0.10	**-0.87**	0.08
Impulsive	0.31	**0.74**	-0.10	-0.01	0.05	**0.54**	**0.52**	-0.06	Low ICC		
Eccentric	0.15	**0.67**	-0.21	0.19	Low Communality				Low ICC		
Unpredictable	0.37	**0.60**	0.02	-0.04	0.05	0.35	**0.55**	-0.26	Low ICC		
Persistent	0.28	**0.47**	0.25	0.27	-0.33	**0.62**	0.18	-0.01	**0.84**	-0.10	0.11
Confident	0.00	0.24	**0.75**	0.20	**-0.75**	0.31	0.07	-0.01	**0.49**	-0.26	-0.37
Strong	-0.05	-0.05	**0.72**	0.32	**-0.52**	0.13	0.33	0.20	Low ICC		
Bold	0.17	0.23	**0.71**	0.21	**-0.64**	0.38	0.13	-0.07	Low ICC		
Effective	0.27	0.26	**0.60**	0.30	**-0.59**	**0.42**	0.18	0.09	**0.95**	0.06	0.06
Direct/forceful/gets own way	0.37	0.32	**0.51**	0.28	**-0.56**	**0.42**	0.28	0.16	**0.89**	0.12	-0.09
Independent	-0.05	**0.40**	**0.47**	0.22	-0.29	0.18	0.32	**0.50**	Low ICC		
Insecure	0.14	-0.24	**-0.61**	0.19	**0.61**	-0.02	-0.12	-0.32	Low ICC		
Cautious	0.04	-0.01	**-0.65**	0.26	**0.74**	-0.25	-0.10	0.00	**-0.52**	0.34	0.27
Timid	-0.12	-0.14	**-0.69**	0.38	**0.84**	-0.10	-0.07	0.08	**-0.68**	0.20	0.23
Nervous/anxious/not calm	0.21	**0.40**	**-0.73**	0.07	**0.79**	0.19	**0.40**	-0.13	0.00	-0.15	**0.82**
Fearful	-0.01	0.05	**-0.88**	0.13	**0.89**	-0.05	0.10	0.11	Low Communality		
Apprehensive	0.08	0.15	**-0.91**	0.17	**0.89**	-0.02	0.08	0.15	-0.13	0.30	**0.67**
Lazy	-0.20	**-0.48**	-0.18	**0.62**	0.27	-0.33	-0.09	**0.61**	0.04	**0.89**	0.04
Slow	-0.30	-0.28	-0.20	**0.61**	-0.06	-0.16	-0.22	**0.72**	0.11	**0.88**	-0.02
Depressed	-0.17	-0.14	**-0.41**	**0.43**	**0.64**	0.02	0.22	**0.51**	-0.02	**0.61**	0.38
Submissive/subordinate	Low ICC				**0.92**	0.08	-0.11	-0.14	**-0.70**	-0.16	0.39
Tense	Low ICC				**0.44**	-0.14	0.37	0.38	-0.31	0.22	**0.55**
Sociable	Low Communality				-0.01	**0.84**	-0.13	-0.05	Low MSA		
Affiliative/Companionable	Low ICC				-0.03	**0.72**	-0.16	-0.03	Low ICC		
Solitary	Low Communality				0.32	**-0.46**	0.05	0.35	Low MSA		
Sensitive	Low ICC				Low Communality				Low ICC		

Several items were also excluded due to low MSA or low communality. For the rhesus macaques, we excluded sociable and solitary due to low communality. For the long-tailed macaques, we excluded eccentric and sensitive, due to low communality. For the bonnet macaques, we excluded understanding and fearful for low communality.

From the original 51 items, we retained 37 in a four-factor rhesus macaque model, 41 in a four-factor long-tailed macaque model, and 19 in a three-factor bonnet macaque model. For the factor names, we appended a superscript for each of the relevant species (‘R’ for rhesus macaques, ‘L’ for long-tailed, and ‘B’ for bonnet) as well as a subscript (_H_ for human situation) to distinguish these factors from the general factors. The rhesus macaque dataset supported a four-factor solution (**Table 5; S13 Table in S1 File**), with the factors explaining 72% of the variation in the data. Factor names and the proportion of variation explained were: Irritable^R^_H_, 0.25; Exploratory^R^_H_, 0.20; Apprehensive^R^_H_, 0.20; and Lazy^R^_H_, 0.07. The long-tailed macaque dataset supported a four-factor solution (**Table 5; S14 Table in S1 File**), with the factors explaining 71% of the variation in the data. Factor names and the proportion of variation explained were: Apprehensive^L^_H_, 0.25; Exploratory^L^_H_, 0.20; Irritable^L^_H_, 0.18; and Lazy^L^_H_, 0.08. The bonnet macaque dataset supported a three-factor solution (**Table 5; S15 Table in S1 File**), with the factors explaining 73% of the variation in the data. Factor names and the proportion of variation explained were: Effective^B^_H_, 0.34; Lazy/Exploratory^B^_H_, 0.25; and Apprehensive^B^_H_, 0.13.

The resulting models had low RMSR, with a degrees of freedom correction (0.05, 0.04, and 0.03 for the bonnet, long-tailed, and rhesus macaque models, respectively), and were below 0.08 indicating acceptable goodness-of-fit. Alpha and total omega estimates were high across the three models (≥ 0.95). Communalities were high, with means of 0.72 ±0.12*sd* for rhesus, 0.71 ±0.13*sd* for long-tailed, and 0.73 ±0.10*sd* for bonnet macaques. Interfactor correlation coefficients are presented in the supplementary materials (**S16 Table in S1 File**). Our model structures relative to item and subject sample sizes were sufficient based on screening recommendations for communality values, item:factor ratios, and sample size [89].

Following our approach with the general models, we screened the human situation factor models to determine if structure was markedly changed with the omission of complex items; simple models were highly correlated (*r* ≥ |0.91|) with full models containing complex items (**S3 Text, S17 Table in S1 File**). Additionally, distinct reliability cut-offs (i.e., ICC[3,1] ≤ 0.10 or ≤ 0.00) did not alter our model structures (**S1 Text; S1 Fig; S4 Table in S1 File**).

#### Species comparisons of human-situation ratings

We compared the factor models between the three species (**Table 6**). We observed high cross-correlations in two factors that showed structural comparability across the three taxa (Apprehensive^RLB^_H_, Exploratory^RL^_H_, and Lazy^RL^_H_ with Lazy/Exploratory^B^ comparable to both of the latter factors) as well as one factor that showed comparability between long-tailed and rhesus macaques (Irritable^RL^), with an average |*r*| between the respective factor loadings of 0.87.

**Table 6:** For the human situation factor models, Tucker’s congruence (Φ) and Pearson’s correlation coefficients (*r*) present in three matrices, each representative of pairwise cross-species comparisons between Procrustes rotated factor loadings.

	Φ	*r*	Φ	*r*	Φ	*r*	Φ	*r*
**Long-tailed by Rhesus macaques**								
	Irritable^R^_H_		Exploratory^R^_H_		Apprehensive^R^_H_		Lazy^R^_H_	
Apprehensive^L^_H_	0.04	0.03	0.33	0.33	0.93	0.93	0.03	-0.06
Exploratory^L^_H_	0.20	0.20	0.87	0.80	0.37	0.44	0.04	-0.71
Irritable^L^_H_	0.95	0.95	0.22	0.15	0.04	0.04	0.01	-0.18
Lazy^L^_H_	0.00	-0.04	0.04	-0.47	0.04	0.03	0.77	0.63
**Long-tailed by Bonnet macaques**								
	Effective^B^_H_		Lazy/Exploratory^B^_H_		Apprehensive^B^_H_			
Apprehensive^L^_H_	-0.21	-0.44	-0.44	-0.57	0.95	0.97		
Exploratory^L^_H_	0.58	0.34	0.92	0.94	-0.49	-0.63		
Irritable^L^_H_	0.82	0.72	0.65	0.57	-0.25	-0.33		
Lazy^L^_H_	0.27	0.07	-0.49	-0.72	0.10	0.09		
**Rhesus by Bonnet macaques**								
	Effective^B^_H_		Lazy/Exploratory^B^_H_		Apprehensive^B^_H_			
Irritable^R^_H_	0.78	0.75	0.56	0.50	0.47	0.47		
Exploratory^R^_H_	0.56	0.33	0.90	0.92	0.41	0.42		
Apprehensive^R^_H_	0.44	0.61	0.38	0.42	0.97	0.98		
Lazy^R^_H_	0.43	-0.09	-0.31	-0.85	-0.03	-0.17		

We found *good* congruence for Irritable^RL^_H_, Apprehensive^RB^_H_, and Apprehensive^LB^_H_ (**Table 6**). We found *fair* congruence for Apprehensive^RL^_H_, Exploratory^RL^_H_, between Lazy/Exploratory^B^_H_ and Exploratory^L^_H_, as well as Lazy/Exploratory^B^_H_ and Exploratory^R^_H_. We also found *poor* congruence between Effective^B^_H_ and Irritable^L^_H_. Effective^B^_H_ was incongruent with all of the rhesus macaque factors. Finally, Lazy^RL^_H_ was incongruent. To determine what items resulted in the greatest contribution to incongruence, we conducted a stepwise removal (**S4 Text in S1 File**). Congruence for Lazy^RL^_H_ was improved by the removal of insecure.

We completed fuzzy set analyses on three factors that were represented across all three species (Apprehensive^RLB^, Exploratory^RL^, Lazy^RL^ with Lazy/Exploratory^B^ comparable to both latter factors) and one factor that was represented across just long-tailed and rhesus macaques (Irritable^RL^) (**Table 7; S18 Table in S1 File**). We appended a superscript ‘F’ to distinguish fuzzy sets from factors. Using a threshold of ≥ |0.40|, we found that across all three species: the Exploratory^F^ fuzzy set included the items active/energetic, affiliative/companionable, curious/exploratory/inquisitive, eccentric, impulsive, opportunistic, sociable, and solitary; the Apprehensive^F^ fuzzy set included the items apprehensive, bold, fearful, insecure, nervous/anxious/not calm, strong, and tense. The Irritable^F^ fuzzy set included the items aggressive, bullying, calm/equable, defiant, equable, excitable, feisty, gentle, irritable, reckless, tolerant, understanding, and unemotional; the Lazy^F^ fuzzy set included the items depressed, lazy, and slow. Thus, we found evidence for similar item placement in each of these four factors.

**Table 7:** Items with scores ≥ |0.40| in our fuzzy set analyses for four factors in our human situation factor model. Items are represented in all species models, unless otherwise indicated with superscript for which items are in each species model (annotated with the superscript of ‘B’ for bonnet, ‘L’ for long-tailed, or ‘R’ for rhesus macaques). See **S18 Table in S1 File** for the remaining items.

Items	Fuzzy Sets			
	Exploratory^F^_H_	Apprehensive^F^_H_	Lazy^F^_H_	Irritable^F^_H_*
Sociable^L^	**0.84**	-0.01	-0.05	-0.13
Curious/exploratory/inquisitive	**0.78**	-0.03	-0.09	-0.01
Active/Energetic	**0.72**	0.08	-0.21	0.08
Affiliative/Companionable^L^	**0.72**	-0.03	-0.03	-0.16
Eccentric^R^	**0.67**	0.21	0.19	0.15
Opportunistic	**0.56**	0.05	0.03	-0.04
Impulsive^LR^	**0.54**	0.05	-0.01	0.31
Solitary^L^	**-0.46**	0.32	0.35	0.05
Fearful^LR^	-0.05	**0.88**	0.11	-0.01
Nervous/anxious/not calm	0.15	**0.73**	0.07	0.21
Apprehensive	-0.02	**0.67**	0.15	0.08
Insecure^LR^	-0.02	**0.61**	0.19	-0.12
Tense^BL^	-0.14	**0.44**	0.22	0.37
Strong^LR^	-0.05	**-0.52**	0.20	-0.05
Bold^LR^	0.23	**-0.64**	-0.07	0.13
Slow	-0.16	-0.02	**0.61**	-0.22
Lazy	-0.33	0.04	**0.61**	-0.09
Depressed	0.02	0.38	**0.43**	-0.17
Irritable^LR^	-0.02	0.00	0.08	**0.89**
Aggressive^LR^	0.09	-0.08	0.14	**0.82**
Feisty^LR^	0.02	-0.05	0.05	**0.80**
Excitable^LR^	0.20	0.01	0.18	**0.66**
Bullying	-0.15	-0.04	0.12	**0.58**
Reckless	0.26	-0.10	-0.07	**0.46**
Defiant	0.20	0.01	0.01	**0.45**
Unemotional^LR^	0.01	0.13	0.29	**-0.51**
Tolerant^LR^	0.19	-0.12	0.17	**-0.55**
Gentle^LR^	-0.05	0.01	0.19	**-0.59**
Understanding^LR^	0.10	-0.10	-0.11	**-0.67**
Equable^LR^	0.18	-0.06	0.20	**-0.69**
Calm/Equable^LR^	-0.02	-0.07	0.23	**-0.76**

*These factors were only present in rhesus and long-tailed macaques.

### Comparing general conspecific and human situation models

After averaging across observers, the reliable item ratings were highly correlated across the general and human situation surveys (correlations were run across subjects within each item; the mean *r* ± *sd* = 0.62 ± 0.14 [rhesus macaques], 0.65 ± 0.16 [long-tailed macaques], and 0.71 ± 0.10 [bonnet macaques]). In the long-tailed macaque ratings, only affiliative/companionable and sociable had Pearson’s correlations < 0.30; similarly, in the rhesus macaque ratings, only sociable fell below the same threshold; in the bonnet macaque ratings, none of the items fell below this threshold (the opportunistic coefficient of *r* = 0.50 was the lowest).

To assess differences between the general factor model and the human situation model, we extracted regression factor scores from and compared subjects’ factor scores between the two factor structures (**Table 8**). Across the three taxa, the Active^RLB^ factors showed moderate-to-high associations with Exploratory^RL^_H_ or Lazy/Exploratory^B^_H_ in human situations (*r* = -0.82 in bonnet, 0.69 in long-tailed, -0.43 in rhesus macaques): more Active monkeys were more Exploratory (note that these correlations are directionally consistent when considering factor item structure, though item loadings of Lazy/Exploratory^B^_H_ and Active^R^ would need to be reversed for the correlation to be positive across all three species). Although, across taxa, the Active^RLB^ factors were moderately correlated (*r* ≥0.30) with all of the human situation factors except Apprehensive^RB^_H_ in rhesus and bonnet macaques as well as Irritable^L^_H_ long-tailed macaques. The Confident^RLB^ factors showed moderate-to-high associations with Apprehensive^RLB^_H_ in human situations (*r* = -0.62 in bonnet, -0.79 in long-tailed, -0.75 in rhesus macaques). In bonnet macaques, however, Confident^B^ was also highly correlated with Effective^B^_H_ in human situations in bonnet macaques (*r* = 0.71). The Irritable^R^, Equable^R^, and Irritable/Equable^L^ factors were highly associated with Irritable^RL^_H_ in human situations for long-tailed (*r* = 0.73) and rhesus macaques (*r* = 0.60 and -0.54 for Irritable^R^ and Equable^R^, respectively). The negative correlation for Equable^R^ was due to opposing loadings between Irritable^R^ and Equable^R^ (e.g., see the item gentle’s loadings). In bonnet macaques, Equable^B^ was moderately associated with Apprehensive^B^_H_ in human situations (*r* = 0.35). Sociable^B^ was moderately-to-highly associated with Apprehensive^B^_H_ (*r* = 0.56) and Lazy/Exploratory^B^_H_ (*r* = -0.46) in human situations for bonnet macaques, but for long-tailed macaques Sociable^L^ was moderately associated with Irritable^L^_H_ in human situations (*r* = -0.31). Sociable^R^ was not associated with any of the human situation factors for rhesus macaques. In rhesus macaques, the Irritable^R^ factor had moderate-to-high correlations with all five of the factors isolated from human situations (*r* = 0.38–0.60). In long-tailed macaques, Playful^L^ was moderately associated with Lazy^L^_H_ in human situations (*r* = 0.47).

**Table 8:** Pearson’s correlation coefficients between general factors (rows) and human situation factors (columns); factors are ordered alphabetically. Significance, uncorrected for multiple comparisons, is annotated with asterisks, such that: * < 0.05; ** < 0.01; *** <0.001.

		Human Situation Factors										
		Rhesus macaques				Long-tailed macaques				Bonnet macaques		
		Apprehensive^R^_H_	Exploratory^R^_H_	Irritable^R^_H_	Lazy^R^_H_	Apprehensive^L^_H_	Exploratory^L^_H_	Irritable^L^_H_	Lazy^L^_H_	Apprehensive^B^_H_	Effective^B^_H_	Lazy/ Exploratory ^B^_H_
**Personality Factors**	Active^RLB^	-0.29***	-0.43***	-0.41***	0.48***	-0.49***	0.69***	0.23*	-0.60***	-0.03	0.37**	-0.82***
	Confident^RLB^	-0.75***	-0.08	0.03	-0.02	-0.79***	0.23*	-0.06	-0.01	-0.62***	0.71***	-0.15
	Irritable^R^	0.39***	0.38***	0.60***	0.39***	–	–	–	–	–	–	–
	Irritable/ Equable^L^	–	–	–	–	0.01	0.08	0.73***	-0.15	–	–	–
	Equable^RLB^	0.08	0.20*	-0.54***	0.40***	–	–	–	–	0.35**	0.23*	-0.05
	Playful^L^	–	–	–	–	-0.29**	0.26**	-0.07	0.47***	–	–	–
	Sociable^RLB^	-0.07	-0.08	-0.01	0.11	-0.06	-0.02	-0.31**	-0.25*	-0.49***	0.17	-0.34**

Comparisons between item compositions in our fuzzy set analyses (**Tables 4** and **7**) showed similarities with the results from our factor score correlations. First, Confident^F^ and Apprehensive^F^_H_ both included apprehensive, insecure, fearful, and nervous/anxious/not calm as salient items. Active^F^ and Lazy^F^_H_ included lazy and slow, but Exploratory^F^_H_ also included active/energetic, curious/exploratory/inquisitive, and impulsive. Sociable^F^ and Exploratory^F^_H_ both included affiliative/companionable, eccentric, sociable, and solitary. Finally, the Irritable^F^_H_ human situation fuzzy set, present only in rhesus and long-tailed macaques, included items associated with Equable^F^ (calm/equable, equable, gentle, and tolerant) as well as Irritable^F^ (bullying, excitable, feisty, and irritable).

## Discussion

Our study provides evidence of the generalizability of personality structure across three group-living wild animal species. When rated by teams of human observers familiar with their behavior, rhesus, long-tailed, and bonnet macaques all evidenced four factors: Confident^RLB^, Sociable^RLB^, Active^RLB^ and Irritable/Equable (which varied in its structure across species). This organization is broadly comparable to those found in rhesus and long-tailed macaques from captive or free-ranging (i.e., naturalistic laboratory) populations [38, 63, 90–93]. Three personality factors, limited to human situations, emerged across all our study: Exploratory^RLB^_H,_ Apprehensive^RLB^_H_, and Lazy^RLB^_H_, though Lazy^RL^_H_ was incongruent. Additionally, both rhesus and long-tailed macaques had an Irritable^RL^_H_ factor.

Our methods were consistent across species, and therefore facilitated cross-species comparability in underrepresented study animals and environments [4]. Consequently, we addressed a critical gap in the literature related to understanding comparative animal personality and individual behavioral differences [23], by assessing both cross-species and cross-situational differences in behavioral components linked to personality. The presence of the same four general personality traits across species (Confident, Sociable, Active, and Irritable/Equable) indicates a core phylogenetic similarity, an important finding among wild populations that are potentially subject to adaptively responding to current conditions, and is similar to outcomes reported with distinct surveys in a comparative survey of six macaque species–which included wild, free-ranging, and captive populations [36]. In line with our expectations for an association between factor structure and social styles, rhesus macaques, the more despotic of the species, exhibited a personality structure more strongly defined by aggression: Irritable^R^ explained the highest proportion of model variation (0.25). Long-tailed macaques had a factor with high loadings in items representing both Irritable and Equable (i.e., Irritable/Equable^L^), while bonnet macaques had a factor more strongly defined by Equable^B^ items.

The human situation factor models supported our expectations both of social style and adaptive response to urbanization: long-tailed and rhesus macaques both exhibited personality structures more strongly defined by aggressive and bold traits as evident via the Irritable^RL^_H_ factor. Thus, in addition to the influences of species-typical evolutionary history or social styles [28, 36], the expression of personality may also be influenced through adaptive behavioral responses to rapidly changing urban or urban-rural environments. Indeed, this is consistent with more recent perspectives of aspects of macaque social structure having been shaped by both evolutionary underpinnings and adaptations to current socio-ecological factors [30, 31]. Disentangling the relative contributions and explanatory power of these evolutionary and adaptive processes would require the consideration of additional macaque groups and/or species, and by applying formal phylogenetic methods [94].

### General conspecific factor models

We found evidence for four structurally equivalent factors across the three species: Confident^RLB^, Sociable^RLB^, Active^RLB^, and an Irritable/Equable complex. This evidence supports similarities in these trait-like structures composed of similar items among these macaque species. For rhesus macaques, our general factor structure was comparable to those reported by Capitanio and colleagues [63, 90, 91] with Confident^R^ similar to Confidence (inclusive of: bold, cautious, direct, effective, fearful, submissive, timid); Sociable^R^ to Sociability (inclusive of: affiliative, warm, sociable, solitary); Equable^R^ with Equability (inclusive of: calm, equable, understanding), and Irritable^R^ with Irritability (inclusive of: irritable) (**S19 Table in S1 File**). Our factor structure differed in that aggressive was placed with Irritable^R^, rather than Confidence (or, here, Confident^R^); similarly, excitable was placed with Irritable^R^, rather than Excitable. Additionally, tense and reckless were absent in our study (due to low communality and ICC, respectively). Tense and reckless loaded on Confidence and Irritability in the Capitanio lab studies, respectively. Finally, we found divergence in Active^R^ (i.e., active/energetic, curious/exploratory/inquisitive, playful, slow as they loaded on Excitable, Sociable/Confident, Sociable, and Equable/Excitable, respectively). This divergence was largely attributable to the absence of Active^R^ as a personality factor in Capitanio and colleagues’ studies. We posit that one possibility for this distinction might be that our subjects were wild while those from the other studies were captive. In support of this assertion, our factors contained similar items as those in dimensions reported in free-ranging rhesus macaques on Cayo Santiago [48]. We found structural equivalence between our Irritable^R^ factor and their Dominance dimension (inclusive of: bullying, stingy/greedy, jealous, aggressive, irritable, defiant, excitable, gentle, manipulative); Confident^R^ and Confidence (inclusive of: fearful, submissive, timid, cautious, anxious); Sociable^R^ and Friendliness (inclusive of: sociable, protective, intelligent, depressed, solitary); and Active^R^ with Activity (inclusive of: active, lazy, and playful). Such equivalence is indicative of consistent structure in rhesus macaques across populations, environments, and protocols.

For long-tailed macaques, our general factor structure was comparable to a model from 34 captive long-tailed macaques [93]. We found structural equivalence between our Confident^L^ factor and their Emotionality (inclusive of: anxious, apprehensive, confident, fearful, insecure, popular, strong); Active^L^ with Activity (inclusive of: active, curious, opportunistic); Sociable^L^ with Sociability (inclusive of: popular, protective, sociable, solitary); and, Equable^L^ with Tolerance (inclusive of: aggressive, equable, understanding). Our factor structure differed in that effective loaded on Confident^L^, rather than Activity. Additionally, playful and strong loaded on Playful^L^, rather than Emotionality [93]. Additionally, in a combined trait adjective and behavior factor model, Uher et al. [92] reported broadly similar factors of: Playful-active-curious, Aggressive-competitive, Prosocial-gregarious, and Assertive-nonanxious. Their use of a survey that included both behavioral descriptive verb and trait adjective items limits the utility of direct comparisons; nevertheless, we found structural equivalence with our Playful^L^ and Active^L^ factors and their reported Playful-active-curious factor (playful, curious, active, impulsive). Such equivalence, while more limited in scope relative to rhesus macaques, is indicative of consistent structure in long-tailed macaques across populations, environments, and protocols.

To our knowledge there is no published study on bonnet macaque personality, so we are not able to establish concordance with previous research for this species. Compared to the other two species we studied, however, our bonnet macaque model lacked a fifth factor–that is, the rhesus macaque model included Irritable^R^ while the long-tailed macaque model included Playful^L^. One explanation for this could be species-typical differences in phylogenetic distances and/or the consequential cross-species differences in social styles. Alternatively, we can examine the factor structure of the long-tailed and rhesus macaques. For instance, as supported by our simplified model that excluded complex items, the omitted Playful^L^ factor in long-tailed macaques could be similar to Active^L^ [92] or Emotionality [93]. Even so, our rhesus macaque model also suggested a five-factor model; thus, the discrepancy in bonnet macaque model structure can likely only be resolved through future work.

We found a progression in the factor compositions among our populations that align with the grades of social style [28, 36]. Long-tailed macaques, as a grade 2 species, had a single Irritable/Equable^L^ factor that represented many of the items present in the rhesus macaque Irritable^R^ factor and Equable^R^ factors. The single bonnet macaque factor, however, was primarily composed of items associated with Equable^B^ as would be expected for a more tolerant grade 3 species. A more expansive survey design with strong *a priori* expectations regarding these structural differences would likely be necessary to elucidate these species differences.

Finally, though we referred to social style ‘grades’, such variance is likely better represented as a continuum of ‘social reaction norms’ in response to the same types of conditions [31, 95]. To better test for a continuum of interspecies differences in personality traits, it would be necessary to include extremely tolerant or egalitarian species (e.g., crested or Tonkean macaques [29, 96]), or nonhuman primate genera that characterize interspecific variation in social tolerance. For example, there are many primate taxa that exhibit variation in social styles among subtaxa (e.g., leaf-eating monkeys [Colobinae], mangabeys [*Lophocebus*; *Rungwecebus*; *Cercocebus*], guenons [*Cercopithecus*], or baboons [*Papio*]) and would, therefore, be suitable for providing additional insight into a structural continuum for social style.

### Human situation factor models

We expected that species might have distinct responses to sympatry with humans or synanthropy, and could expect particularly salient trait expression among species in dense anthropogenic environments and/or with more frequent human interactions. Human situation models supported the existence of four factors: Exploratory^RLB^_H,_ Lazy^RLB^_H,_ and Apprehensive^RLB^_H_; and, Irritable^RL^_H_ in long-tailed and rhesus macaques. Many items in Irritable^RL^_H_ (aggressive, calm/equable, irritable, impulsive, gentle, feisty, excitable, equable, tolerant, unemotional, understanding) were omitted in bonnet macaques due to low reliability. We also note that Effective^B^_H_ in the bonnet macaque model contained several items that loaded on Apprehensive^RL^_H_ (cautious, confident, and timid); other items, however, in Apprehensive^RL^_H_ were excluded due to low reliability or communality scores in the bonnet macaque model (e.g., bold, insecure, fearful, strong).

The reduced reliability in the human situation ratings for bonnet macaques on items that loaded on Irritable^RL^_H_ could potentially be explained by differences in behavior that has species-typical or socio-ecological underpinnings. For instance, this population of bonnet macaques has much lower rates of human-directed aggression events, relative to the rhesus and long-tailed macaques [12]. These lower instances are inter-related to the less urban locale that the bonnet macaques occupied. Thus, the absence of Irritable likely reflects behavioral characteristics, unique either to this particular bonnet macaque population that experiences low levels of anthropogenic impact, or to bonnet macaques more generally [35, 97, 98]. Disentangling these differences awaits the future assessments of intraspecific cross-population differences of human situation personality ratings.

While comparable survey designs of human situation ratings are scarce, our and others’ work indicates that there are traits that are salient in human situations. For instance, we found similar factor structure to that reported by Pritchard et al. [42]. Their raters frequently observed subjects on man-made platforms with frequent human-animal interactions [99–101]. Our Apprehensive^F^_H_ fuzzy set resembled factors of Insecurity (apprehensive, fearful, insecure, submissive, tense), Exploratory^F^_H_ to Boldness (aggressive, equable, excitable, gentle, irritable), and Irritable^F^_H_ to Reactivity (active, curious). The human intruder test [102] isolated Activity (which includes exploratory behaviors), Emotionality (which includes fear-associated behaviors), and Aggression as salient response factors in 5000 infant rhesus macaques [102, 103]. Finally, our Apprehensive^F^_H_, Irritable^F^_H_, and Exploratory^F^_H_ fuzzy sets show similarities to Fearfulness, Aggressiveness, and Cautiousness components, respectively, from behavioral responses to human tests [47].

While parallels between these studies suggest similarities among traits relevant to human-interactions, these items may not be distinguishable from general ratings despite somewhat distinct factor structure. For example, Baker et al. [37] isolated a Human–Sociability factor in Barbary macaques, but this factor was composed of both human-animal items (nervous, cooperative, cautious, and social) as well as general items (allogrooming, flexible, and effective). Similarly, our Exploratory^F^_H_ fuzzy set included items associated with the Sociable^F^ fuzzy set. Namely, affiliative/companionable, sociable, and solitary. Importantly, in the human situation ratings, these items are markers of prosocial behaviors expressed to humans rather than to conspecifics. Whether the Exploratory^F^_H_ factor offers insight into how macaques respond to humans requires future research. Such personality trait associations would interact with the effects of other known socio-demographic qualities’ influences on human engagement [12, 13].

### Comparisons between general and human situation models

We found similar structures in a set of general and human situation factors. For instance, Active^RLB^ was associated with the Exploratory^RLB^_H_ factors and shared similar items. Dammhahn [104], conducting open field and novel object tests mouse lemurs (*Microcebus murinus*), reported similar results whereby activity, exploration, and boldness were posited to be linked in a behavioral syndrome, i.e. behavioral traits correlated and coupled across situations [105]. Thus, if we assume a behavioral syndrome between Active^RLB^ and Exploratory^RLB^_H_, then Active-like traits could be used to anticipate how macaques would interact with humans as a management tool. This interpretation, however, is challenging to reconcile with the absence of an Active trait in personality rating studies on captive rhesus macaques that regularly interacted with humans [63]; this discrepancy might parallel the absence of a pure Active trait in our human situation results.

We posit that Apprehensive and Confident are so structurally similar that they are likely to be functionally equivalent factors across situations. The Confident^RLB^ factor scores were correlated with the human situation factor scores of Apprehensive^RLB^_H_ and Effective^B^_H_. Additionally, Confident^RB^ and Apprehensive^RLB^_H_ factor loadings and fuzzy sets shared many of the same items. This proposition is not unfounded given that the stress response system is highly conserved [106, 107], that Boldness is a broadly quantified trait across challenging contexts [1], and that Boldness is important to fitness across contexts [108].

In restricting our focus to the rhesus and long-tailed models, the Irritable/Equable complex was strongly associated with the Irritable^RL^_H_ factor. These are likely to be functionally similar given that both factors shared high loadings and fuzzy set contributions from several items. This interpretation, however, does not incorporate the knowledge that the general Irritable^R^ factor was moderately associated with all of the human situation factors in rhesus macaques. Sussman et al. [47] reported a similar occurrence, with aggressive behaviors contributing to all components resulting from cage-side behavioral coding. As previously emphasized, rhesus macaques are generally expected to be despotic [28, 29] with demonstrated covariation in their personality structure [36].

Finally, we isolated a Lazy^RL^_H_ factor in rhesus and long-tailed macaques, and a Lazy/Exploratory^B^_H_ factor in bonnet macaques. While Lazy^RL^_H_ was incongruent, three items exhibited high factor loadings in both species, and mean loading scores were similar–as evident with the Pearson’s correlation coefficients. The three items that composed Lazy^F^_H_ (lazy, slow, depressed) do not strictly require the subject to interact with extrinsic stimuli as opposed to some of the items contained within Exploratory^F^_H_. Given Active was associated with Exploratory_H_ and Lazy_H_, we posit that these two factors reflect differences in assessment based on an individual’s active response to extrinsic stimuli (Exploratory_H_) or overall behavior (Lazy_H_). Resolving these factors by including similar items would improve clarity, as well, as to the incongruence with Lazy^RL^_H_.

### Limitations and further considerations

Our study is not without limitations. First, behavioral nuances may not have been directly observable to the human raters who collected data, as raters were distinct across sites although trained extensively on the same tool by the same trainer (EBM). In other words, the infrequent and homogenous interactions that our bonnet macaques had with humans [12] could also be attributed to raters’ limited capacity to effectively obtain a strong impression of how individuals vary in their responsivity to humans. Furthermore, our item and subject sample size, relative to the number of factors, was more limited for the bonnet macaques.

Second, inter-observer cultural differences might have impacted personality ratings. For instance, observers who contributed to our project were diverse in national and cultural identity. In humans, cultural variation in stereotypes can skew raters’ expectations [109]. We do not dispute the validity of ratings and the capacity for these ratings to measure personality ratings of nonhumans, without being instantiated as anthropomorphic projections [38]. Rather, raters might inadvertently introduce cultural framings or biases–though standardized training and reliability are expected to mitigate such effects. Such care in assessment design would facilitate parsing out whether items were not reliable due to stereotypes, errors in interpretation of the traits or definitions, and/or a psychobiological lack of variation or expression of that trait in the subject.

## Conclusion

Differences and similarities across the three species’ factor models parallel distinctions in social style grades. Future research would benefit from expanding the taxa studied to accommodate covariation that exists in anthropogenic presence and phylogenetic relatedness. Furthermore, executing distinct surveys that emphasize varied responses to situations in wild complex systems would be key for determining which general traits exhibit cross-situational stability versus situation-specific variance. Such approaches are important, not just in the context of comparative personality among primates, but also in understanding individual-, population-, and species-level differences in tolerance of human sympatry.

## Supporting information

S1 File Supplementary materials for personality trait structures across three species of *Macaca*, using survey ratings of responses to conspecifics and humans. Contains all supplementary text, tables, and figures.
(PDF)
