# Supplementary material for: Personality trait structures across three species of Macaca, using survey ratings of responses to conspecifics and humans
Source: PLoS One. 2024 Sep 6;19(9):e0309946. doi: 10.1371/journal.pone.0309946 (PMC11379396; doi:10.1371/journal.pone.0309946)
Supplement: S1 File — Contains all supplementary text, tables, and figures. (PDF) [file pone.0309946.s001.pdf]

**Supplementary Materials**

Personality trait structures across three species of *Macaca*, using survey ratings of responses to conspecifics and humans.

Alexander J. Pritchard[1,2]*, Eliza Bliss-Moreau[1,3], Krishna N. Balasubramaniam[2,4], John P. Capitanio[1], Pascal R. Marty[2,5], Stefano S. K. Kaburu[2,6], Małgorzata E. Arlet[7], Brianne A. Beisner[1], & Brenda McCowan[1,2]

[1] California National Primate Research Center, University of California Davis, Davis, CA, United States of America

[2] Department of Population Health & Reproduction, School of Veterinary Medicine, University of California Davis, Davis, CA, United States of America

[3] Department of Psychology, University of California Davis, Davis, CA, United States of America

[4] School of Life Sciences, Faculty of Science & Engineering, Anglia Ruskin University, Cambridge, United Kingdom

[5] Nature Reserve and Wildlife Park Goldau, Goldau, Switzerland

[6] School of Animal, Rural and Environmental Sciences, Nottingham Trent University, Nottingham, United Kingdom

[7] Institute of Human Biology and Evolution, Faculty of Biology, Adam Mickiewicz University, Poznań, Poland

* Corresponding author

E-mail: ajpritchard@ucdavis.edu (AJP)

**S1 Table.** ICC (3,k) results, with unreliable values in bold: ICC(3,k) < 0.40.

| | Bonnet | | | Long-tailed | | | Rhesus | | |
|---|---|---|---|---|---|---|---|---|---|
| **Item** | **Lower** | **ICC** | **Upper** | **Lower** | **ICC** | **Upper** | **Lower** | **ICC** | **Upper** |
| Active/ Energetic | 0.69 | 0.78 | 0.85 | 0.73 | 0.80 | 0.86 | 0.72 | 0.78 | 0.83 |
| Affiliative/ Companionable | 0.75 | 0.83 | 0.88 | 0.78 | 0.84 | 0.88 | 0.56 | 0.66 | 0.74 |
| Aggressive | 0.64 | 0.74 | 0.82 | 0.53 | 0.66 | 0.75 | 0.71 | 0.77 | 0.82 |
| Apprehensive | 0.41 | 0.58 | 0.71 | 0.69 | 0.77 | 0.84 | 0.64 | 0.72 | 0.79 |
| Bold | 0.62 | 0.73 | 0.81 | 0.79 | 0.84 | 0.89 | 0.73 | 0.79 | 0.84 |
| Bullying | 0.70 | 0.78 | 0.85 | 0.46 | 0.60 | 0.71 | 0.66 | 0.73 | 0.79 |
| Calm/Equable | 0.20 | 0.43 | 0.61 | 0.47 | 0.61 | 0.72 | 0.53 | 0.63 | 0.72 |
| Cautious | 0.56 | 0.69 | 0.79 | 0.68 | 0.77 | 0.83 | 0.50 | 0.61 | 0.70 |
| Confident | 0.70 | 0.78 | 0.85 | 0.76 | 0.82 | 0.87 | 0.77 | 0.82 | 0.86 |
| Curious/ exploratory/ inquisitive | 0.70 | 0.79 | 0.86 | 0.29 | 0.48 | 0.62 | 0.30 | 0.45 | 0.58 |
| Defiant | 0.59 | 0.71 | 0.80 | 0.27 | 0.46 | 0.61 | 0.48 | 0.60 | 0.69 |
| Depressed | 0.58 | 0.70 | 0.79 | 0.60 | 0.70 | 0.79 | 0.49 | 0.60 | 0.69 |
| Direct/ forceful/ gets own way | 0.66 | 0.76 | 0.84 | 0.82 | 0.87 | 0.91 | 0.79 | 0.84 | 0.87 |
| Eccentric | 0.43 | 0.59 | 0.72 | 0.56 | 0.68 | 0.77 | 0.40 | 0.53 | 0.64 |
| Effective | 0.64 | 0.75 | 0.83 | 0.75 | 0.81 | 0.87 | 0.82 | 0.86 | 0.89 |
| Equable | 0.25 | 0.47 | 0.63 | 0.46 | 0.60 | 0.72 | 0.56 | 0.66 | 0.74 |
| Excitable | 0.43 | 0.59 | 0.72 | 0.17 | **0.39** | 0.56 | 0.45 | 0.57 | 0.67 |
| Fearful | -0.11 | **0.21** | 0.46 | 0.72 | 0.79 | 0.85 | 0.72 | 0.78 | 0.83 |
| Feisty | 0.59 | 0.70 | 0.80 | 0.48 | 0.62 | 0.73 | 0.52 | 0.62 | 0.71 |
| Gentle | 0.23 | 0.45 | 0.62 | 0.64 | 0.74 | 0.81 | 0.52 | 0.63 | 0.71 |
| Impulsive | 0.52 | 0.66 | 0.77 | 0.35 | 0.52 | 0.66 | 0.40 | 0.53 | 0.64 |
| Independent | -0.25 | **0.11** | 0.39 | 0.62 | 0.72 | 0.80 | 0.67 | 0.74 | 0.80 |
| Insecure | 0.03 | **0.31** | 0.52 | 0.35 | 0.53 | 0.66 | 0.42 | 0.55 | 0.65 |
| Intelligent | 0.30 | 0.50 | 0.66 | 0.19 | 0.41 | 0.58 | 0.29 | 0.44 | 0.57 |
| Irritable | 0.48 | 0.63 | 0.74 | 0.41 | 0.56 | 0.69 | 0.60 | 0.69 | 0.76 |
| Jealous | 0.61 | 0.72 | 0.81 | 0.31 | 0.49 | 0.64 | 0.56 | 0.65 | 0.73 |
| Lazy | 0.68 | 0.78 | 0.85 | 0.57 | 0.68 | 0.77 | 0.78 | 0.83 | 0.87 |
| Manipulative | 0.45 | 0.61 | 0.73 | 0.52 | 0.65 | 0.75 | 0.45 | 0.57 | 0.67 |
| Nervous/ anxious/ not calm | 0.56 | 0.69 | 0.78 | 0.31 | 0.49 | 0.64 | 0.53 | 0.63 | 0.72 |
| Nurturant | 0.26 | 0.47 | 0.64 | 0.83 | 0.87 | 0.91 | 0.64 | 0.72 | 0.78 |
| Opportunistic | 0.46 | 0.61 | 0.73 | 0.40 | 0.56 | 0.69 | 0.41 | 0.53 | 0.64 |
| Persistent | 0.52 | 0.66 | 0.77 | 0.52 | 0.65 | 0.75 | 0.45 | 0.57 | 0.67 |
| Playful | 0.90 | 0.93 | 0.95 | 0.68 | 0.77 | 0.83 | 0.63 | 0.71 | 0.78 |
| Popular | 0.74 | 0.82 | 0.87 | 0.86 | 0.90 | 0.93 | 0.69 | 0.76 | 0.81 |
| Protective | 0.68 | 0.77 | 0.84 | 0.77 | 0.83 | 0.88 | 0.67 | 0.74 | 0.80 |
| Reckless | 0.61 | 0.72 | 0.81 | 0.59 | 0.70 | 0.78 | -0.06 | **0.17** | 0.36 |
| Sensitive | -0.06 | **0.24** | 0.48 | -0.05 | **0.23** | 0.45 | -0.05 | **0.18** | 0.37 |
| Slow | 0.60 | 0.71 | 0.80 | 0.29 | 0.48 | 0.63 | 0.72 | 0.78 | 0.83 |
| Sociable | 0.74 | 0.81 | 0.87 | 0.82 | 0.87 | 0.91 | 0.62 | 0.70 | 0.77 |
| Solitary | 0.73 | 0.80 | 0.87 | 0.81 | 0.86 | 0.90 | 0.59 | 0.68 | 0.75 |
| Stingy/ greedy | 0.30 | 0.50 | 0.66 | 0.63 | 0.72 | 0.80 | 0.47 | 0.58 | 0.68 |
| Strong | 0.59 | 0.71 | 0.80 | 0.83 | 0.87 | 0.91 | 0.85 | 0.88 | 0.91 |
| Submissive/ subordinate | 0.71 | 0.80 | 0.86 | 0.80 | 0.86 | 0.90 | 0.68 | 0.75 | 0.81 |
| Tense | 0.57 | 0.69 | 0.79 | 0.58 | 0.69 | 0.78 | 0.36 | 0.50 | 0.61 |
| Timid | 0.41 | 0.58 | 0.71 | 0.72 | 0.79 | 0.85 | 0.61 | 0.70 | 0.77 |
| Tolerant | 0.11 | **0.37** | 0.56 | 0.61 | 0.71 | 0.79 | 0.57 | 0.67 | 0.74 |
| Understanding | 0.32 | 0.52 | 0.67 | 0.32 | 0.50 | 0.64 | 0.33 | 0.47 | 0.60 |
| Unemotional | 0.29 | 0.50 | 0.65 | 0.53 | 0.66 | 0.75 | 0.05 | **0.26** | 0.43 |
| Unpredictable | 0.37 | 0.55 | 0.69 | -0.01 | **0.26** | 0.47 | 0.35 | 0.49 | 0.61 |
| Vigilant | 0.14 | **0.39** | 0.58 | 0.43 | 0.58 | 0.70 | 0.00 | **0.21** | 0.40 |
| Warm/ affectionate | 0.61 | 0.72 | 0.81 | 0.82 | 0.87 | 0.91 | 0.58 | 0.67 | 0.75 |

**S2 Table.** ICC (3,1) results, with unreliable values in bold: ICC(3,k) < 0.40.

| | Bonnet | | | Long-tailed | | | Rhesus | | |
|---|---|---|---|---|---|---|---|---|---|
| **Item** | **Lower** | **ICC** | **Upper** | **Lower** | **ICC** | **Upper** | **Lower** | **ICC** | **Upper** |
| Active/ Energetic | 0.30 | 0.41 | 0.52 | 0.40 | 0.50 | 0.60 | 0.34 | 0.42 | 0.50 |
| Affiliative/ Companionable | 0.38 | 0.49 | 0.59 | 0.47 | 0.56 | 0.65 | 0.20 | 0.28 | 0.36 |
| Aggressive | 0.26 | 0.36 | 0.48 | 0.22 | 0.32 | 0.43 | 0.33 | 0.40 | 0.48 |
| Apprehensive | 0.12 | 0.22 | 0.33 | 0.35 | 0.45 | 0.56 | 0.27 | 0.34 | 0.42 |
| Bold | 0.24 | 0.35 | 0.46 | 0.48 | 0.57 | 0.67 | 0.35 | 0.43 | 0.51 |
| Bullying | 0.31 | 0.42 | 0.53 | 0.17 | 0.27 | 0.39 | 0.28 | 0.35 | 0.43 |
| Calm/Equable | 0.05 | 0.13 | 0.24 | 0.18 | 0.28 | 0.39 | 0.18 | 0.25 | 0.34 |
| Cautious | 0.21 | 0.31 | 0.42 | 0.35 | 0.45 | 0.55 | 0.17 | 0.24 | 0.32 |
| Confident | 0.32 | 0.42 | 0.53 | 0.44 | 0.54 | 0.63 | 0.40 | 0.47 | 0.55 |
| Curious/ exploratory/ inquisitive | 0.32 | 0.43 | 0.54 | 0.09 | 0.18 | 0.29 | 0.08 | 0.14 | 0.21 |
| Defiant | 0.23 | 0.33 | 0.45 | 0.08 | 0.18 | 0.29 | 0.16 | 0.23 | 0.31 |
| Depressed | 0.21 | 0.32 | 0.43 | 0.27 | 0.37 | 0.48 | 0.16 | 0.23 | 0.31 |
| Direct/ forceful/ gets own way | 0.28 | 0.39 | 0.51 | 0.53 | 0.62 | 0.71 | 0.43 | 0.50 | 0.58 |
| Eccentric | 0.13 | 0.22 | 0.34 | 0.24 | 0.35 | 0.46 | 0.12 | 0.18 | 0.26 |
| Effective | 0.27 | 0.37 | 0.49 | 0.43 | 0.52 | 0.62 | 0.47 | 0.54 | 0.62 |
| Equable | 0.06 | 0.15 | 0.26 | 0.17 | 0.27 | 0.39 | 0.20 | 0.28 | 0.36 |
| Excitable | 0.13 | 0.23 | 0.34 | 0.05 | **0.14** | 0.25 | 0.14 | 0.21 | 0.29 |
| Fearful | -0.02 | **0.05** | 0.14 | 0.39 | 0.49 | 0.59 | 0.34 | 0.42 | 0.50 |
| Feisty | 0.22 | 0.32 | 0.44 | 0.19 | 0.29 | 0.40 | 0.18 | 0.25 | 0.33 |
| Gentle | 0.06 | 0.14 | 0.25 | 0.31 | 0.41 | 0.52 | 0.18 | 0.25 | 0.33 |
| Impulsive | 0.18 | 0.28 | 0.40 | 0.12 | 0.21 | 0.32 | 0.12 | 0.18 | 0.26 |
| Independent | -0.04 | **0.02** | 0.11 | 0.29 | 0.39 | 0.50 | 0.29 | 0.37 | 0.45 |
| Insecure | 0.01 | **0.08** | 0.18 | 0.12 | 0.22 | 0.33 | 0.13 | 0.20 | 0.27 |
| Intelligent | 0.08 | 0.17 | 0.28 | 0.06 | 0.15 | 0.25 | 0.08 | 0.14 | 0.21 |
| Irritable | 0.15 | 0.25 | 0.37 | 0.15 | 0.24 | 0.36 | 0.23 | 0.31 | 0.39 |
| Jealous | 0.24 | 0.34 | 0.46 | 0.10 | 0.20 | 0.31 | 0.20 | 0.27 | 0.36 |
| Lazy | 0.30 | 0.41 | 0.52 | 0.25 | 0.35 | 0.46 | 0.42 | 0.49 | 0.57 |
| Manipulative | 0.14 | 0.24 | 0.35 | 0.21 | 0.31 | 0.42 | 0.14 | 0.21 | 0.29 |
| Nervous/ anxious/ not calm | 0.20 | 0.30 | 0.42 | 0.10 | 0.19 | 0.30 | 0.18 | 0.26 | 0.34 |
| Nurturant | 0.07 | 0.15 | 0.26 | 0.55 | 0.63 | 0.72 | 0.26 | 0.34 | 0.42 |
| Opportunistic | 0.14 | 0.24 | 0.36 | 0.14 | 0.24 | 0.35 | 0.12 | 0.19 | 0.26 |
| Persistent | 0.18 | 0.28 | 0.40 | 0.21 | 0.32 | 0.43 | 0.14 | 0.21 | 0.29 |
| Playful | 0.65 | 0.73 | 0.80 | 0.35 | 0.45 | 0.55 | 0.25 | 0.33 | 0.41 |
| Popular | 0.37 | 0.47 | 0.58 | 0.61 | 0.69 | 0.76 | 0.31 | 0.38 | 0.46 |
| Protective | 0.30 | 0.40 | 0.52 | 0.45 | 0.55 | 0.64 | 0.29 | 0.36 | 0.45 |
| Reckless | 0.24 | 0.34 | 0.46 | 0.26 | 0.36 | 0.47 | -0.01 | **0.04** | 0.10 |
| Sensitive | -0.01 | **0.06** | 0.16 | -0.01 | **0.07** | 0.17 | -0.01 | **0.04** | 0.10 |
| Slow | 0.23 | 0.33 | 0.45 | 0.09 | 0.19 | 0.30 | 0.34 | 0.41 | 0.50 |
| Sociable | 0.36 | 0.47 | 0.58 | 0.54 | 0.63 | 0.71 | 0.24 | 0.32 | 0.40 |
| Solitary | 0.35 | 0.45 | 0.56 | 0.52 | 0.61 | 0.69 | 0.22 | 0.30 | 0.38 |
| Stingy/ greedy | 0.08 | 0.17 | 0.28 | 0.29 | 0.40 | 0.50 | 0.15 | 0.22 | 0.30 |
| Strong | 0.22 | 0.33 | 0.44 | 0.55 | 0.64 | 0.72 | 0.54 | 0.61 | 0.67 |
| Submissive/ subordinate | 0.33 | 0.44 | 0.55 | 0.51 | 0.60 | 0.69 | 0.30 | 0.37 | 0.46 |
| Tense | 0.21 | 0.31 | 0.43 | 0.26 | 0.36 | 0.47 | 0.10 | 0.16 | 0.24 |
| Timid | 0.12 | 0.21 | 0.33 | 0.39 | 0.49 | 0.59 | 0.24 | 0.32 | 0.40 |
| Tolerant | 0.02 | **0.10** | 0.21 | 0.28 | 0.38 | 0.49 | 0.21 | 0.28 | 0.37 |
| Understanding | 0.09 | 0.18 | 0.29 | 0.10 | 0.20 | 0.31 | 0.09 | 0.15 | 0.23 |
| Unemotional | 0.08 | 0.16 | 0.27 | 0.22 | 0.32 | 0.43 | 0.01 | **0.07** | 0.13 |
| Unpredictable | 0.11 | 0.20 | 0.31 | 0.00 | **0.08** | 0.18 | 0.10 | 0.16 | 0.24 |
| Vigilant | 0.03 | **0.11** | 0.22 | 0.16 | 0.26 | 0.37 | 0.00 | **0.05** | 0.12 |
| Warm/ affectionate | 0.24 | 0.34 | 0.46 | 0.54 | 0.62 | 0.71 | 0.22 | 0.29 | 0.37 |

*S1 Text. Comparisons of ICC cut-offs*

We recognize the diversity of cut-offs that have been used in studies of primate personality. Relative to ICC(3,k), ICC(3,1) is more conservative in the magnitude of its estimates [1]. Furthermore, though they are mathematically similar in their calculation [2–4], their interpretation differs [4]. Thus, we also present our results informed by cut-offs with ICC(3,1). ICC(3,1) share similarities with Pearson's correlation coefficients, albeit with an emphasis on additivity not linearity [4]. This is an important and relevant point as guidelines on effect sizes for correlational studies of individual differences suggest that small correlations might be reasonably expected to be 0.10. We note that our study's cut-off of ICC(3,k) ≤ 0.40 is similar to an ICC(3,1) cut-off of 0.10 (**S1 Figure**). Indeed, the mean ICC(3,1) of discarded items with our ICC(3,k) cut off of 0.40 were: 0.07 ±0.03*sd* and 0.06 ±0.04*sd*, for the general and human-situation ratings, respectively. We recognize that a wide variety of studies utilize an ICC reliability cut-off of 0.00 [5–7]. Thus, we replicated our analyses as to how our factor structure does or does not change with these two additional thresholds (ICC[3,1] 0.10 and 0.00). We present summaries of these factor models here.

**S1 Figure.** Comparisons of our ICC(3,k) (x-axis) and ICC(3,1) (y-axis) estimates for (A) the general surveys and (B) the human-situation surveys. We have added lines at ICC(3,k) = 0.40 (vertical red) and ICC(3,1) (horizontal blue). Note that our cut-off of ICC(3,k) = 0.40 is broadly similar to an ICC(3,1) of 0.10. None of our items had ICC(3,1) < 0.00; sensitive$^{R}_{H}$ was 0.00 for both ICC estimates.

A)

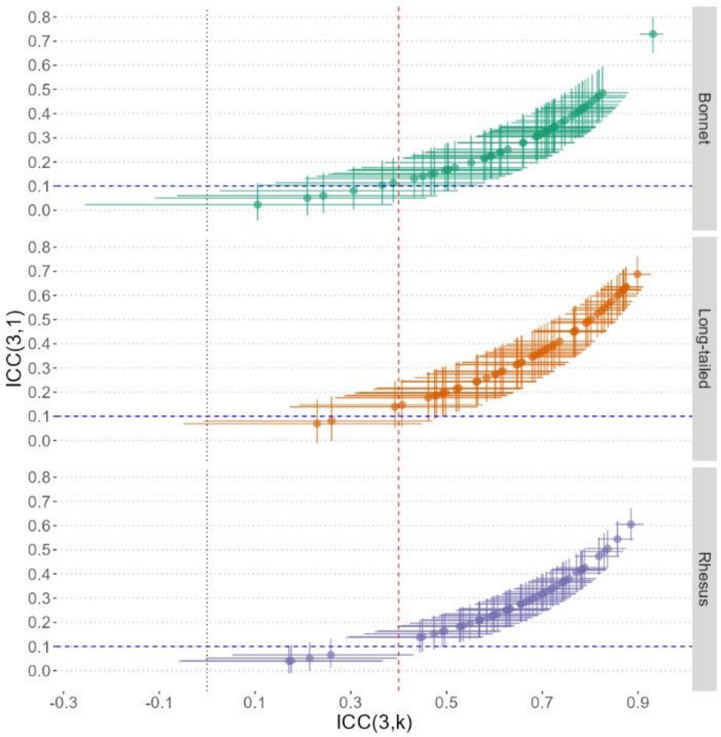


B)

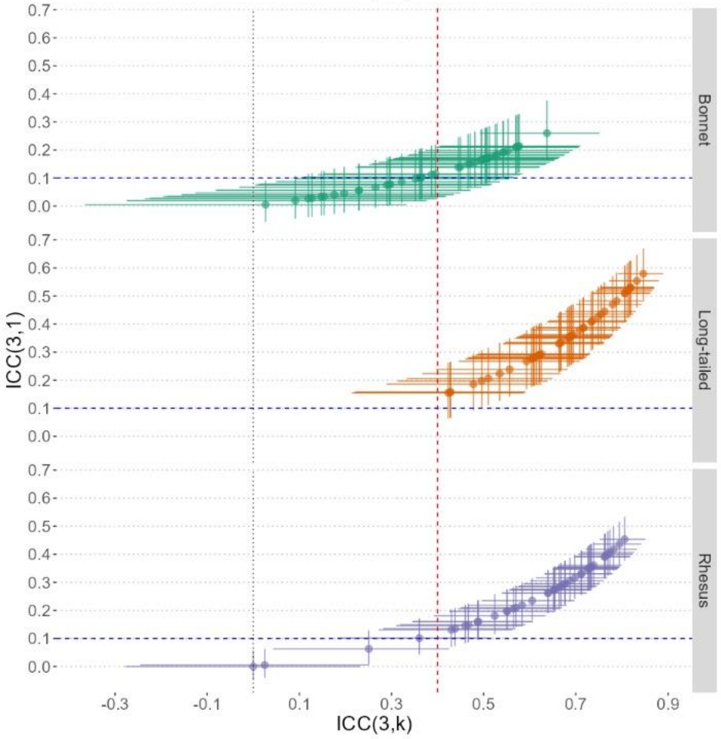

An ICC(3,1) of 0.10 would yield very similar results to those reported in the manuscript, with identical or near identical factor structures and the addition of only 1-4 items in four of the six models (**S3 Table**). With an ICC(3,1) threshold of 0.00, we obtained similar results for the general survey factor models across all species with an additional 2-3 items (**S4 Table**). For the human-situation factor models, we obtained also similar results in the rhesus and long-tailed macaque with an ICC(3,1) threshold of 0.00. For the bonnet macaque human-situation model, however, our ICC(3,1) 0.00 threshold model explained less variation than our other human-situation models (**S4 Table**). Even so, we found 3 factors with similar factor loadings ($r \geq |0.993|$) and good congruence ($\Phi \geq |0.99|$) to our model reported in the manuscript. A fourth factor emerged relative to our reported model: $\text{Equable}^{B}_{H}$. This factor was correlated ($r = 0.761$), but not congruent ($\Phi = 0.74$), with $\text{Lazy/Exploratory}^{B}_{H}$. The items that composed this factor, however, were all low in ICC(3,1): 0.066 ±0.049*sd*, with a range of 0.005 to 0.140; the highest loading items had ICC(3,1) estimates of 0.034 and 0.005. We report this here as a potential factor for future interest, but retain our threshold for consistency with the other species and models. We also reinforce, as reported in the manuscript, that the bonnet macaques were pronounced in having generally low reliability in the human situation, an observation that we have generated hypotheses from relevant for future research.

**S3 Table.** Comparing general factor models with varying reliability cut-offs.

| **ICC cut-off for item exclusion** | ICC(3,k) ≤ 0.40 | | | ICC(3,1) ≤ 0.10 | | | ICC(3,1) ≤ 0.00 | | |
|---|---|---|---|---|---|---|---|---|---|
| **Species** | Rhesus | Long-Tailed | Bonnet | Rhesus | Long-Tailed | Bonnet | Rhesus | Long-Tailed | Bonnet |
| **Number of Items excluded by ICC** | 4 | 3 | 6 | 4 | 2 | 4 | 0 | 0 | 0 |
| **Number of items retained in model*** | 45 | 45 | 45 | 45 | 46 | 46 | 47 | 47 | 48 |
| **Number of factors** | 5 | 5 | 4 | 5 | 5 | 4 | 5 | 5 | 4 |
| **Range of \|*r*\| for item factor loadings relative to the ICC(3,k) ≤ 0.40 model**** | NA | NA | NA | 1.000 to 1.000 | 1.000 to 1.000 | 1.000 to 0.999 | 1.000 to 1.000 | 1.000 to 1.000 | 1.000 to 0.998 |
| **Range of Φ for item factor loadings relative to the ICC(3,k) ≤ 0.40 model**** | NA | NA | NA | 1.00 to 1.00 | 1.00 to 1.00 | 1.00 to 1.00 | 1.00 to 1.00 | 1.00 to 1.00 | 1.00 to 1.00 |
| **Cumulative Variation Explained** | 73% | 76% | 75% | 73% | 76% | 75% | 72% | 76% | 74% |

*After MSA and communality screening procedures described in the manuscript

**The model in the manuscript is the ICC(3,k) model presented here. As the order of the factors can switch, we correlated factors based on similar item composition not order of variance explained. For example, Irritable/Equable$^{L}$ and Sociable$^{L}$ both explained 0.16 of the proportional variance and their factor order in the model often switched.

**S4 Table.** Comparing human-situation factor models with varying reliability cut-offs.

| **ICC cut-off for item exclusion** | ICC(3,k) ≤ 0.40 | | | ICC(3,1) ≤ 0.10 | | | ICC(3,1) ≤ 0.00 | | |
|---|---|---|---|---|---|---|---|---|---|
| **Species** | Rhesus | Long-Tailed | Bonnet | Rhesus | Long-Tailed | Bonnet | Rhesus | Long-Tailed | Bonnet |
| **Number of Items excluded by ICC** | 4 | 0 | 22 | 3 | 0 | 16 | 0 | 0 | 0 |
| **Number of items retained in the model*** | 37 | 41 | 19 | 38 | 41 | 23 | 40 | 41 | 37 |
| **Number of factors** | 4 | 4 | 3 | 4 | 4 | 3 | 4 | 4 | 4 |
| **Range of \|*r*\| for item factor loadings relative to the ICC(3,k) ≤ 0.40 model**** | – | – | – | 1.000 to 1.000 | 1.000 to 1.000 | 0.997 to 0.942 | 1.000 to 0.998 | 1.000 to 1.000 | 0.993 to 0.761† |
| **Range of \|Φ\| for item factor loadings relative to the ICC(3,k) ≤ 0.40 model**** | NA | NA | NA | 1.00 to 1.00 | 1.00 to 1.00 | 1.00 to 0.92 | 1.00 to 1.00 | 1.00 to 1. 00 | 1.00 to 0.74† |
| **Cumulative Variation Explained** | 72% | 71% | 73% | 72% | 71% | 71% | 70% | 71% | 66% |

*After MSA and communality screening procedures described in the manuscript

**The model in the manuscript is the ICC(3,k) model presented here. As the order of the factors can switch, we correlated factors based on similar item composition not order of variance explained.

† Three of the four factor loadings were highly correlated (*r* ≥ |0.993|) and highly congruent with our reported models (Φ ≥ |0.99|). One additional factor, however, was found which was defined by items with low reliability. See discussion above for details.

**S5 Table.** Rhesus macaque factor structure for general personality ratings. Bolded items exceed a loading of |0.40|.

| | Factors | | | | |
|---|---|---|---|---|---|
| **Items** | Irritable | Confident | Sociable | Active | Equable |
| Irritable | **0.84** | -0.01 | -0.06 | -0.02 | -0.22 |
| Bullying | **0.79** | -0.19 | 0.03 | -0.06 | -0.08 |
| Excitable | **0.77** | 0.08 | 0.04 | -0.15 | -0.09 |
| Jealous | **0.77** | -0.13 | 0.18 | 0.00 | -0.12 |
| Stingy/greedy | **0.75** | -0.13 | 0.05 | 0.14 | -0.11 |
| Feisty | **0.73** | -0.06 | 0.11 | -0.18 | -0.15 |
| Unpredictable | **0.70** | 0.31 | -0.19 | -0.27 | 0.12 |
| Aggressive | **0.67** | -0.30 | -0.01 | -0.05 | -0.17 |
| Persistent | **0.64** | -0.21 | 0.15 | -0.02 | 0.04 |
| Defiant | **0.62** | 0.00 | 0.22 | -0.17 | 0.01 |
| Impulsive | **0.54** | 0.18 | -0.19 | **-0.47** | 0.27 |
| Opportunistic | **0.44** | -0.13 | 0.19 | -0.23 | 0.20 |
| Fearful | 0.03 | **0.91** | -0.02 | 0.09 | 0.06 |
| Apprehensive | 0.08 | **0.91** | 0.11 | 0.10 | 0.02 |
| Nervous/anxious/not calm | 0.34 | **0.85** | 0.04 | -0.06 | 0.02 |
| Insecure | 0.12 | **0.75** | 0.37 | 0.10 | -0.12 |
| Submissive/subordinate | -0.14 | **0.70** | -0.22 | 0.11 | 0.27 |
| Timid | -0.14 | **0.69** | -0.03 | 0.26 | 0.17 |
| Cautious | -0.13 | **0.68** | -0.09 | 0.20 | 0.18 |
| Protective | 0.21 | **-0.53** | 0.38 | 0.15 | 0.24 |
| Intelligent | 0.09 | **-0.54** | 0.18 | -0.25 | **0.43** |
| Bold | **0.54** | **-0.58** | 0.06 | 0.00 | 0.11 |
| Direct/forceful/ gets own way | **0.48** | **-0.63** | 0.15 | 0.06 | 0.04 |
| Confident | **0.40** | **-0.66** | 0.13 | -0.02 | 0.10 |
| Strong | 0.29 | **-0.68** | -0.22 | 0.14 | 0.33 |
| Effective | 0.36 | **-0.72** | 0.19 | 0.13 | 0.03 |
| Sociable | 0.18 | 0.00 | **0.89** | 0.06 | 0.07 |
| Affiliative/Companionable | 0.14 | 0.10 | **0.88** | 0.09 | 0.15 |
| Warm/affectionate | 0.01 | 0.13 | **0.79** | 0.01 | 0.36 |
| Nurturant | -0.08 | 0.07 | **0.71** | 0.03 | 0.35 |
| Popular | 0.11 | -0.38 | **0.68** | -0.08 | 0.11 |
| Manipulative | **0.48** | -0.01 | **0.54** | -0.05 | -0.17 |
| Independent | 0.28 | **-0.43** | **-0.47** | -0.05 | 0.39 |
| Depressed | 0.12 | 0.26 | **-0.56** | **0.49** | 0.24 |
| Solitary | 0.17 | 0.14 | **-0.72** | 0.13 | **0.45** |
| Lazy | 0.06 | 0.10 | -0.04 | **0.90** | 0.11 |
| Slow | -0.01 | 0.04 | -0.04 | **0.86** | 0.16 |
| Curious/exploratory/inquisitive | 0.04 | 0.00 | 0.01 | **-0.63** | **0.50** |
| Active/Energetic | 0.25 | -0.05 | -0.18 | **-0.78** | 0.18 |
| Tolerant | -0.30 | 0.10 | 0.30 | 0.11 | **0.65** |
| Equable | -0.34 | -0.08 | 0.13 | 0.22 | **0.65** |
| Understanding | -0.19 | -0.21 | 0.35 | -0.03 | **0.65** |
| Calm/Equable | -0.33 | -0.09 | 0.13 | 0.35 | **0.61** |
| Playful | -0.18 | 0.10 | 0.11 | **-0.42** | **0.58** |
| Gentle | **-0.41** | 0.12 | 0.32 | 0.15 | **0.57** |

**S6 Table.** Long-tailed macaque factor structure for general personality ratings. Bolded items exceed a loading of |0.40|.

| | Factors | | | | |
|---|---|---|---|---|---|
| **Items** | Confident | Sociable | Irritable/Equable | Active | Playful |
| Confident | **0.90** | 0.06 | -0.01 | 0.01 | 0.11 |
| Bold | **0.82** | 0.03 | 0.11 | 0.06 | 0.18 |
| Direct/forceful/gets own way | **0.81** | 0.11 | 0.19 | -0.02 | 0.21 |
| Effective | **0.78** | 0.18 | 0.12 | 0.08 | 0.16 |
| Stingy/greedy | **0.58** | -0.12 | 0.46 | 0.10 | 0.22 |
| Strong | **0.54** | -0.04 | 0.14 | 0.10 | **0.46** |
| Depressed | **-0.62** | -0.30 | 0.08 | -0.32 | 0.30 |
| Nervous/anxious/not calm | **-0.74** | -0.05 | 0.31 | 0.05 | 0.10 |
| Insecure | **-0.75** | **0.44** | 0.21 | -0.06 | -0.04 |
| Cautious | **-0.81** | -0.02 | 0.00 | -0.20 | 0.04 |
| Timid | **-0.83** | -0.05 | -0.01 | -0.15 | 0.07 |
| Fearful | **-0.83** | -0.12 | 0.08 | -0.15 | 0.02 |
| Apprehensive | **-0.89** | -0.05 | 0.04 | -0.14 | 0.13 |
| Submissive/subordinate | **-0.96** | -0.06 | -0.05 | 0.15 | 0.01 |
| Nurturant | -0.18 | **0.91** | -0.09 | -0.03 | 0.11 |
| Warm/affectionate | 0.02 | **0.91** | -0.10 | 0.00 | 0.06 |
| Sociable | 0.18 | **0.87** | -0.03 | 0.08 | -0.01 |
| Affiliative/companionable | 0.20 | **0.82** | -0.09 | 0.11 | 0.03 |
| Protective | 0.20 | **0.75** | 0.17 | -0.04 | 0.20 |
| Popular | **0.46** | **0.67** | -0.03 | -0.04 | 0.11 |
| Solitary | **-0.43** | **-0.75** | -0.04 | -0.04 | 0.26 |
| Independent | 0.31 | **-0.80** | -0.15 | 0.00 | **0.41** |
| Irritable | -0.04 | 0.03 | **0.95** | -0.12 | 0.04 |
| Feisty | 0.12 | 0.03 | **0.84** | 0.09 | 0.08 |
| Aggressive | **0.40** | 0.10 | **0.70** | 0.07 | 0.23 |
| Bullying | 0.36 | 0.05 | **0.66** | 0.10 | 0.24 |
| Jealous | 0.33 | 0.22 | **0.54** | 0.06 | 0.32 |
| Unemotional | **0.40** | -0.10 | **-0.53** | -0.26 | 0.26 |
| Tolerant | -0.10 | **0.59** | **-0.54** | 0.08 | 0.25 |
| Gentle | -0.28 | **0.50** | **-0.57** | -0.06 | 0.25 |
| Understanding | 0.13 | 0.23 | **-0.64** | 0.09 | 0.03 |
| Equable | 0.29 | 0.15 | **-0.76** | -0.16 | 0.22 |
| Calm/equable | 0.27 | 0.14 | **-0.80** | -0.15 | 0.17 |
| Active/energetic | 0.03 | -0.09 | -0.03 | **0.88** | 0.10 |
| Curious/exploratory/inquisitive | 0.08 | 0.05 | -0.10 | **0.70** | 0.29 |
| Opportunistic | 0.08 | 0.12 | 0.12 | **0.64** | 0.26 |
| Impulsive | -0.05 | -0.06 | **0.43** | **0.55** | 0.13 |
| Reckless | -0.01 | -0.02 | 0.25 | **0.54** | 0.21 |
| Persistent | 0.24 | 0.13 | 0.26 | **0.48** | 0.35 |
| Defiant | 0.14 | 0.15 | 0.37 | 0.38 | 0.21 |
| Tense | -0.27 | -0.13 | **0.40** | **-0.55** | 0.10 |
| Lazy | -0.22 | -0.10 | 0.01 | **-0.75** | 0.31 |
| Slow | 0.17 | 0.13 | -0.09 | **-0.79** | 0.29 |
| Playful | -0.15 | 0.24 | -0.16 | 0.39 | **0.53** |
| Manipulative | 0.11 | 0.31 | 0.31 | 0.25 | 0.34 |

**S7 Table.** Bonnet macaque factor structure for general personality ratings. Bolded items exceed a loading of |0.40|.

| | Factors | | | |
|---|---|---|---|---|
| **Items** | Confident | Active | Sociable | Equable |
| Effective | **0.95** | -0.10 | 0.03 | 0.03 |
| Direct/forceful/ gets own way | **0.92** | -0.10 | 0.07 | -0.01 |
| Strong | **0.87** | -0.06 | **-0.44** | -0.15 |
| Aggressive | **0.87** | -0.13 | -0.06 | 0.34 |
| Bullying | **0.81** | -0.13 | 0.01 | **0.45** |
| Defiant | **0.80** | 0.19 | 0.13 | 0.08 |
| Persistent | **0.77** | 0.23 | 0.02 | 0.12 |
| Protective | **0.77** | -0.22 | 0.26 | -0.09 |
| Confident | **0.77** | 0.26 | 0.18 | -0.21 |
| Bold | **0.74** | **0.42** | -0.02 | -0.11 |
| Manipulative | **0.72** | -0.10 | 0.16 | 0.23 |
| Jealous | **0.66** | -0.06 | 0.27 | **0.44** |
| Feisty | **0.64** | 0.19 | -0.16 | **0.49** |
| Intelligent | **0.59** | -0.01 | 0.24 | -0.31 |
| Stingy/greedy | **0.58** | -0.12 | 0.16 | 0.18 |
| Popular | **0.58** | 0.14 | **0.50** | -0.02 |
| Apprehensive | **-0.52** | -0.23 | -0.35 | 0.37 |
| Tense | **-0.62** | -0.26 | -0.19 | 0.36 |
| Timid | **-0.73** | -0.23 | -0.20 | 0.23 |
| Cautious | **-0.78** | -0.38 | 0.04 | 0.12 |
| Submissive/subordinate | **-0.87** | 0.16 | -0.15 | 0.10 |
| Playful | -0.30 | **0.95** | -0.12 | -0.14 |
| Curious/exploratory/inquisitive | 0.18 | **0.87** | -0.04 | -0.16 |
| Active/Energetic | 0.00 | **0.85** | 0.15 | 0.06 |
| Excitable | -0.16 | **0.70** | 0.04 | **0.47** |
| Reckless | **0.44** | **0.62** | -0.34 | 0.19 |
| Impulsive | 0.25 | **0.57** | -0.31 | **0.40** |
| Opportunistic | 0.36 | **0.43** | 0.27 | 0.23 |
| Depressed | -0.20 | **-0.55** | **-0.52** | 0.01 |
| Lazy | -0.01 | **-0.78** | -0.34 | -0.08 |
| Slow | 0.06 | **-0.80** | -0.30 | -0.14 |
| Warm/affectionate | 0.01 | -0.10 | **0.81** | -0.13 |
| Affiliative/Companionable | 0.19 | 0.20 | **0.80** | 0.03 |
| Nurturant | -0.09 | -0.18 | **0.80** | -0.10 |
| Sociable | 0.24 | 0.23 | **0.76** | 0.03 |
| Understanding | 0.03 | -0.24 | **0.59** | -0.35 |
| Unemotional | -0.10 | -0.34 | **-0.47** | -0.40 |
| Unpredictable | 0.21 | **0.45** | **-0.49** | 0.33 |
| Eccentric | 0.12 | -0.06 | **-0.63** | 0.13 |
| Solitary | -0.15 | -0.15 | **-0.81** | -0.14 |
| Irritable | **0.41** | 0.00 | -0.06 | **0.66** |
| Nervous/anxious/not calm | **-0.51** | 0.06 | -0.22 | **0.62** |
| Gentle | **-0.45** | 0.09 | 0.04 | **-0.65** |
| Calm/Equable | -0.02 | -0.25 | 0.06 | **-0.68** |
| Equable | 0.20 | -0.08 | 0.04 | **-0.81** |

**S8 Table.** Interfactor correlation coefficients (Phi) for the personality factor models.

**Rhesus macaques**

| | Irritable$^{R}$ | Confident$^{R}$ | Sociable$^{R}$ | Active$^{R}$ |
|---|---|---|---|---|
| Confident$^{R}$ | -0.35 | – | – | – |
| Sociable$^{R}$ | 0.04 | -0.17 | – | – |
| Active$^{R}$ | -0.32 | 0.17 | 0.01 | – |
| Equable$^{R}$ | -0.15 | -0.02 | 0.14 | 0.12 |

**Long-tailed macaques**

| | Confident$^{L}$ | Sociable$^{L}$ | Irritable/Equable$^{L}$ | Active$^{L}$ |
|---|---|---|---|---|
| Sociable$^{L}$ | 0.29 | – | – | – |
| Irritable/Equable$^{L}$ | 0.08 | -0.15 | – | – |
| Active$^{L}$ | 0.33 | 0.19 | 0.25 | – |
| Playful$^{L}$ | 0.31 | 0.16 | 0.07 | 0.07 |

**Bonnet macaques**

| | Confident$^{B}$ | Active$^{B}$ | Sociable$^{B}$ |
|---|---|---|---|
| Active$^{B}$ | 0.25 | | |
| Sociable$^{B}$ | 0.29 | 0.15 | |
| Equable$^{B}$ | 0.15 | 0.17 | -0.20 |

*S2 Text. Comparing Complex Item General Models, versus Simple Models*

Due to the number of complex items (i.e., items with moderate-to-heavy loadings across multiple factors), we constructed simple models to determine whether these complex items altered model structure. Following recommendations by Howard [8], simple models met the criteria that all items loaded on at least one factor ≥ 0.40, did not exceed a loading of 0.30 for remaining factors, and exceeded 0.20 between the maximum loading and the next highest loading. We excluded items one-by-one that failed this criterion and reassessed the number of factors, as detailed in the methods, with the stipulation that we did not decrease the number of factors below 4, to retain comparability between the models. We used Procrustes rotation on the factor loadings and compared congruence between the simplified and complex models. For each of the species, factors from the simple models were highly congruent ($\Phi \geq 0.99$) with the original models (S9 Table), despite a reduction of 13, 15, and 19 items in the simplified bonnet, long-tailed, and rhesus macaque models, respectively. The original number of factors were retained in the bonnet macaque model. In the simplified long-tailed macaque model, however, $\text{Playful}^{L}$ was absent, as the two high loading items were eliminated for being complex (playful, independent). In the simplified rhesus macaque model, $\text{Equable}^{R}$ was absent as all the high loading items were eliminated for being complex. We prioritized retaining complex item models to increase comparability between the three species, as their removal reduced the number of items represented across species' models.

**S9 Table.** Congruence between factors from the personality factor models with complex items included or excluded. Simplified factors, from models with complex items are excluded, are italicized.

**Rhesus macaques**

| | Irritable$^{R}$ | Confident$^{R}$ | Sociable$^{R}$ | Active$^{R}$ | Equable$^{R}$ |
|---|---|---|---|---|---|
| *Irritable*$^{R}$ | 0.99 | -0.16 | 0.16 | -0.12 | -0.43 |
| *Confident*$^{R}$ | -0.17 | 1.00 | -0.05 | 0.15 | 0.23 |
| *Sociable*$^{R}$ | 0.16 | -0.05 | 0.99 | 0.02 | 0.51 |
| *Active*$^{R}$ | -0.13 | 0.14 | 0.02 | 1.00 | 0.20 |

**Long-tailed macaques**

| | Confident$^{L}$ | Sociable$^{L}$ | Irritable/ Equable$^{L}$ | Active$^{L}$ | Playful$^{L}$ |
|---|---|---|---|---|---|
| *Confident*$^{L}$ | 0.99 | 0.15 | -0.03 | 0.14 | 0.33 |
| *Sociable*$^{L}$ | 0.15 | 1.00 | -0.08 | 0.06 | 0.19 |
| *Irritable/Equable*$^{L}$ | -0.03 | -0.08 | 1.00 | 0.10 | 0.07 |
| *Active*$^{L}$ | 0.15 | 0.06 | 0.11 | 1.00 | -0.02 |

**Bonnet macaques**

| | Confident$^{B}$ | Active$^{B}$ | Sociable$^{B}$ | Equable$^{B}$ |
|---|---|---|---|---|
| *Confident*$^{B}$ | 1.00 | 0.07 | 0.14 | 0.14 |
| *Active*$^{B}$ | 0.07 | 1.00 | 0.11 | 0.06 |
| *Sociable*$^{B}$ | 0.14 | 0.11 | 1.00 | -0.13 |
| *Equable*$^{B}$ | 0.14 | 0.06 | -0.13 | 0.99 |

**S10 Table.** Items with scores < |0.40| in our fuzzy set analyses for five factors that appeared across our general factor models. See **Table 2** for the remaining items.

| | **Factors** | | | | |
|---|---|---|---|---|---|
| **Items** | Confident | Sociable | Active | Irritable | Equable |
| Aggressive | 0.30 | -0.01 | 0.05 | 0.34 | 0.17 |
| Defiant | 0.00 | 0.13 | 0.17 | 0.08 | -0.01 |
| Depressed | -0.20 | -0.30 | -0.32 | 0.01 | 0.01 |
| Manipulative | 0.01 | 0.16 | 0.05 | 0.23 | 0.17 |
| Opportunistic | 0.08 | 0.12 | 0.23 | 0.12 | 0.12 |
| Persistent | 0.21 | 0.02 | 0.02 | 0.12 | -0.04 |
| Playful | -0.10 | 0.11 | 0.39 | -0.14 | -0.14 |
| Protective | 0.20 | 0.26 | -0.04 | -0.09 | -0.09 |
| Stingy/greedy | 0.13 | 0.05 | 0.10 | 0.18 | 0.11 |
| Tense | -0.27 | -0.13 | -0.26 | 0.36 | 0.36 |
| Understanding | 0.03 | 0.23 | 0.03 | -0.19 | -0.35 |
| Unemotional | -0.10 | -0.10 | -0.26 | -0.40 | -0.40 |
| Unpredictable | 0.21 | -0.19 | 0.27 | 0.33 | -0.12 |

**S11 Table.** ICC (3,k) results for human situation ratings, with unreliable values in bold (ICC < 0.40; lower bound < 0.00).

| | Bonnet | | | Long-tailed | | | Rhesus | | |
|---|---|---|---|---|---|---|---|---|---|
| **Item** | **Lower** | **ICC** | **Upper** | **Lower** | **ICC** | **Upper** | **Lower** | **ICC** | **Upper** |
| Active/Energetic | 0.26 | 0.47 | 0.64 | 0.61 | 0.72 | 0.80 | 0.63 | 0.71 | 0.78 |
| Affiliative/Companionable | 0.09 | **0.35** | 0.56 | 0.22 | 0.43 | 0.59 | 0.04 | **0.25** | 0.42 |
| Aggressive | 0.01 | **0.29** | 0.52 | 0.70 | 0.78 | 0.84 | 0.75 | 0.81 | 0.85 |
| Apprehensive | 0.33 | 0.52 | 0.67 | 0.64 | 0.73 | 0.81 | 0.35 | 0.49 | 0.61 |
| Bold | 0.15 | **0.40** | 0.59 | 0.74 | 0.81 | 0.87 | 0.74 | 0.79 | 0.84 |
| Bullying | 0.37 | 0.55 | 0.69 | 0.58 | 0.69 | 0.78 | 0.70 | 0.76 | 0.82 |
| Calm/Equable | -0.37 | **0.03** | 0.33 | 0.58 | 0.69 | 0.78 | 0.67 | 0.74 | 0.80 |
| Cautious | 0.40 | 0.57 | 0.71 | 0.71 | 0.79 | 0.85 | 0.27 | 0.43 | 0.56 |
| Confident | 0.40 | 0.57 | 0.71 | 0.77 | 0.83 | 0.88 | 0.65 | 0.73 | 0.79 |
| Curious/exploratory/inquisitive | 0.49 | 0.64 | 0.75 | 0.55 | 0.67 | 0.76 | 0.66 | 0.73 | 0.80 |
| Defiant | 0.27 | 0.48 | 0.64 | 0.57 | 0.68 | 0.77 | 0.45 | 0.57 | 0.67 |
| Depressed | 0.31 | 0.51 | 0.66 | 0.48 | 0.62 | 0.73 | 0.35 | 0.49 | 0.61 |
| Direct/forceful/gets own way | 0.31 | 0.51 | 0.66 | 0.75 | 0.82 | 0.87 | 0.58 | 0.67 | 0.75 |
| Eccentric | 0.00 | **0.29** | 0.51 | 0.46 | 0.60 | 0.72 | 0.31 | 0.46 | 0.59 |
| Effective | 0.30 | 0.50 | 0.66 | 0.75 | 0.82 | 0.87 | 0.63 | 0.71 | 0.78 |
| Equable | -0.20 | **0.15** | 0.42 | 0.31 | 0.50 | 0.64 | 0.59 | 0.68 | 0.75 |
| Excitable | -0.23 | **0.12** | 0.40 | 0.33 | 0.51 | 0.65 | 0.55 | 0.65 | 0.73 |
| Fearful | 0.32 | 0.51 | 0.67 | 0.68 | 0.76 | 0.83 | 0.54 | 0.64 | 0.72 |
| Feisty | 0.11 | **0.37** | 0.56 | 0.67 | 0.76 | 0.82 | 0.70 | 0.76 | 0.82 |
| Gentle | -0.13 | **0.20** | 0.45 | 0.22 | 0.43 | 0.59 | 0.60 | 0.69 | 0.76 |
| Impulsive | -0.08 | **0.23** | 0.47 | 0.58 | 0.69 | 0.78 | 0.65 | 0.73 | 0.79 |
| Independent | -0.16 | **0.18** | 0.43 | 0.47 | 0.61 | 0.72 | 0.66 | 0.73 | 0.79 |
| Insecure | -0.08 | **0.23** | 0.47 | 0.45 | 0.59 | 0.71 | 0.39 | 0.52 | 0.63 |
| Intelligent | -0.27 | **0.09** | 0.38 | 0.49 | 0.63 | 0.73 | 0.71 | 0.77 | 0.82 |
| Irritable | 0.10 | **0.36** | 0.56 | 0.54 | 0.66 | 0.76 | 0.71 | 0.77 | 0.83 |
| Lazy | 0.40 | 0.57 | 0.70 | 0.57 | 0.69 | 0.78 | 0.56 | 0.65 | 0.74 |
| Nervous/anxious/not calm | 0.22 | 0.45 | 0.62 | 0.55 | 0.67 | 0.76 | 0.47 | 0.58 | 0.68 |
| Opportunistic | 0.25 | 0.47 | 0.63 | 0.57 | 0.69 | 0.78 | 0.72 | 0.78 | 0.83 |
| Persistent | 0.29 | 0.50 | 0.65 | 0.61 | 0.72 | 0.80 | 0.57 | 0.66 | 0.74 |
| Reckless | 0.34 | 0.53 | 0.68 | 0.75 | 0.82 | 0.87 | 0.56 | 0.65 | 0.73 |
| Sensitive | -0.22 | **0.13** | 0.40 | 0.21 | 0.42 | 0.59 | -0.28 | **0.00** | 0.23 |
| Slow | 0.29 | 0.50 | 0.65 | 0.37 | 0.53 | 0.67 | 0.54 | 0.64 | 0.72 |
| Sociable | 0.14 | **0.39** | 0.58 | 0.47 | 0.61 | 0.72 | 0.43 | 0.55 | 0.66 |
| Solitary | 0.11 | **0.37** | 0.56 | 0.64 | 0.74 | 0.81 | 0.28 | 0.44 | 0.57 |
| Strong | 0.10 | **0.36** | 0.56 | 0.79 | 0.85 | 0.89 | 0.66 | 0.73 | 0.79 |
| Submissive/subordinate | 0.41 | 0.58 | 0.71 | 0.66 | 0.75 | 0.82 | 0.18 | **0.36** | 0.51 |
| Tense | 0.36 | 0.54 | 0.69 | 0.60 | 0.71 | 0.79 | -0.25 | **0.02** | 0.25 |
| Timid | 0.36 | 0.54 | 0.69 | 0.74 | 0.81 | 0.86 | 0.43 | 0.55 | 0.65 |
| Tolerant | -0.03 | **0.27** | 0.50 | 0.74 | 0.81 | 0.86 | 0.61 | 0.70 | 0.77 |
| Understanding | 0.23 | 0.45 | 0.62 | 0.48 | 0.62 | 0.73 | 0.44 | 0.56 | 0.67 |
| Unemotional | 0.05 | **0.32** | 0.54 | 0.48 | 0.61 | 0.72 | 0.32 | 0.47 | 0.59 |
| Unpredictable | -0.19 | **0.15** | 0.42 | 0.40 | 0.56 | 0.68 | 0.50 | 0.61 | 0.70 |
| Vigilant | 0.01 | **0.30** | 0.52 | 0.29 | 0.48 | 0.63 | 0.59 | 0.68 | 0.75 |

**S12 Table.** ICC (3,1) results for human situation ratings, with unreliable values in bold: ICC(3,k) < 0.40.

| | Bonnet | | | Long-tailed | | | Rhesus | | |
|---|---|---|---|---|---|---|---|---|---|
| **Item** | **Lower** | **ICC** | **Upper** | **Lower** | **ICC** | **Upper** | **Lower** | **ICC** | **Upper** |
| Active/Energetic | 0.06 | 0.15 | 0.26 | 0.28 | 0.39 | 0.50 | 0.26 | 0.33 | 0.41 |
| Affiliative/Companionable | 0.02 | **0.10** | 0.20 | 0.07 | 0.16 | 0.27 | 0.01 | **0.06** | 0.13 |
| Aggressive | 0.00 | **0.08** | 0.18 | 0.37 | 0.47 | 0.57 | 0.38 | 0.45 | 0.53 |
| Apprehensive | 0.09 | 0.18 | 0.29 | 0.31 | 0.41 | 0.51 | 0.10 | 0.16 | 0.24 |
| Bold | 0.03 | **0.12** | 0.22 | 0.42 | 0.52 | 0.62 | 0.36 | 0.44 | 0.52 |
| Bullying | 0.11 | 0.20 | 0.31 | 0.25 | 0.36 | 0.47 | 0.32 | 0.39 | 0.47 |
| Calm/Equable | -0.06 | **0.01** | 0.09 | 0.26 | 0.36 | 0.47 | 0.29 | 0.36 | 0.44 |
| Cautious | 0.12 | 0.21 | 0.33 | 0.38 | 0.48 | 0.58 | 0.07 | 0.13 | 0.20 |
| Confident | 0.12 | 0.21 | 0.32 | 0.46 | 0.55 | 0.65 | 0.27 | 0.35 | 0.43 |
| Curious/exploratory/inquisitive | 0.16 | 0.26 | 0.38 | 0.23 | 0.33 | 0.45 | 0.28 | 0.36 | 0.44 |
| Defiant | 0.07 | 0.16 | 0.27 | 0.25 | 0.35 | 0.46 | 0.14 | 0.21 | 0.29 |
| Depressed | 0.08 | 0.17 | 0.28 | 0.19 | 0.29 | 0.40 | 0.10 | 0.16 | 0.24 |
| Direct/forceful/gets own way | 0.08 | 0.17 | 0.28 | 0.43 | 0.53 | 0.63 | 0.21 | 0.29 | 0.37 |
| Eccentric | 0.00 | **0.08** | 0.17 | 0.18 | 0.28 | 0.39 | 0.08 | 0.15 | 0.22 |
| Effective | 0.08 | 0.17 | 0.28 | 0.43 | 0.53 | 0.62 | 0.26 | 0.33 | 0.41 |
| Equable | -0.03 | **0.03** | 0.12 | 0.10 | 0.20 | 0.31 | 0.22 | 0.29 | 0.38 |
| Excitable | -0.04 | **0.03** | 0.12 | 0.11 | 0.21 | 0.32 | 0.20 | 0.27 | 0.35 |
| Fearful | 0.08 | 0.17 | 0.28 | 0.34 | 0.44 | 0.55 | 0.19 | 0.26 | 0.34 |
| Feisty | 0.02 | **0.10** | 0.21 | 0.33 | 0.44 | 0.54 | 0.31 | 0.39 | 0.47 |
| Gentle | -0.02 | **0.05** | 0.14 | 0.07 | 0.16 | 0.26 | 0.23 | 0.31 | 0.39 |
| Impulsive | -0.02 | **0.06** | 0.15 | 0.26 | 0.36 | 0.47 | 0.27 | 0.35 | 0.43 |
| Independent | -0.03 | **0.04** | 0.13 | 0.18 | 0.28 | 0.39 | 0.28 | 0.35 | 0.43 |
| Insecure | -0.02 | **0.06** | 0.15 | 0.17 | 0.27 | 0.38 | 0.11 | 0.18 | 0.26 |
| Intelligent | -0.05 | **0.02** | 0.11 | 0.19 | 0.29 | 0.41 | 0.32 | 0.40 | 0.48 |
| Irritable | 0.02 | **0.10** | 0.20 | 0.23 | 0.33 | 0.44 | 0.33 | 0.41 | 0.49 |
| Lazy | 0.12 | 0.21 | 0.32 | 0.25 | 0.35 | 0.46 | 0.20 | 0.28 | 0.36 |
| Nervous/anxious/not calm | 0.05 | 0.14 | 0.25 | 0.23 | 0.33 | 0.44 | 0.15 | 0.22 | 0.30 |
| Opportunistic | 0.06 | 0.15 | 0.26 | 0.25 | 0.35 | 0.46 | 0.34 | 0.42 | 0.50 |
| Persistent | 0.08 | 0.16 | 0.27 | 0.28 | 0.39 | 0.50 | 0.21 | 0.28 | 0.37 |
| Reckless | 0.09 | 0.18 | 0.30 | 0.43 | 0.53 | 0.63 | 0.20 | 0.27 | 0.36 |
| Sensitive | -0.04 | **0.03** | 0.12 | 0.06 | 0.15 | 0.26 | -0.05 | **0.00** | 0.06 |
| Slow | 0.08 | 0.16 | 0.27 | 0.13 | 0.22 | 0.33 | 0.19 | 0.26 | 0.34 |
| Sociable | 0.03 | **0.11** | 0.22 | 0.18 | 0.28 | 0.39 | 0.13 | 0.20 | 0.28 |
| Solitary | 0.02 | **0.10** | 0.21 | 0.31 | 0.41 | 0.52 | 0.07 | 0.13 | 0.21 |
| Strong | 0.02 | **0.10** | 0.20 | 0.49 | 0.58 | 0.67 | 0.28 | 0.35 | 0.44 |
| Submissive/subordinate | 0.12 | 0.21 | 0.33 | 0.32 | 0.43 | 0.53 | 0.04 | **0.10** | 0.17 |
| Tense | 0.10 | 0.19 | 0.30 | 0.28 | 0.38 | 0.49 | -0.04 | **0.01** | 0.06 |
| Timid | 0.10 | 0.19 | 0.30 | 0.41 | 0.51 | 0.61 | 0.13 | 0.20 | 0.28 |
| Tolerant | -0.01 | **0.07** | 0.16 | 0.41 | 0.51 | 0.61 | 0.24 | 0.32 | 0.40 |
| Understanding | 0.06 | 0.14 | 0.25 | 0.19 | 0.29 | 0.40 | 0.14 | 0.21 | 0.29 |
| Unemotional | 0.01 | **0.09** | 0.19 | 0.18 | 0.29 | 0.40 | 0.09 | 0.15 | 0.22 |
| Unpredictable | -0.03 | **0.04** | 0.13 | 0.14 | 0.24 | 0.35 | 0.16 | 0.23 | 0.32 |
| Vigilant | 0.00 | **0.08** | 0.18 | 0.09 | 0.19 | 0.30 | 0.22 | 0.29 | 0.38 |

**S13 Table.** Rhesus macaque factor structure for human situation ratings. Bolded items exceed a loading of |0.40|.

| | Factors | | | |
|---|---|---|---|---|
| **Items** | Irritable | Exploratory | Apprehensive | Lazy |
| Irritable | **0.92** | -0.02 | 0.00 | 0.27 |
| Aggressive | **0.82** | 0.11 | 0.08 | 0.17 |
| Excitable | **0.81** | 0.20 | -0.01 | 0.18 |
| Feisty | **0.80** | 0.24 | 0.05 | 0.17 |
| Bullying | **0.69** | 0.32 | 0.21 | 0.13 |
| Reckless | **0.65** | 0.26 | 0.25 | 0.25 |
| Defiant | **0.45** | 0.26 | 0.37 | 0.27 |
| Unemotional | **-0.68** | 0.13 | -0.13 | 0.29 |
| Understanding | **-0.75** | **0.43** | 0.10 | 0.11 |
| Tolerant | **-0.82** | 0.19 | 0.16 | 0.25 |
| Gentle | **-0.83** | -0.05 | -0.01 | 0.27 |
| Calm/Equable | **-0.86** | -0.02 | 0.07 | 0.29 |
| Equable | **-0.93** | 0.18 | 0.06 | 0.20 |
| Curious/exploratory/inquisitive | -0.17 | **0.86** | 0.10 | -0.09 |
| Vigilant | 0.05 | **0.84** | -0.20 | -0.01 |
| Intelligent | -0.37 | **0.80** | 0.23 | -0.05 |
| Opportunistic | -0.04 | **0.80** | 0.17 | 0.03 |
| Active/Energetic | 0.12 | **0.76** | 0.18 | -0.21 |
| Impulsive | 0.31 | **0.74** | -0.10 | -0.01 |
| Eccentric | 0.15 | **0.67** | -0.21 | 0.19 |
| Unpredictable | 0.37 | **0.60** | 0.02 | -0.04 |
| Persistent | 0.28 | **0.47** | 0.25 | 0.27 |
| Confident | 0.00 | 0.24 | **0.75** | 0.20 |
| Strong | -0.05 | -0.05 | **0.72** | 0.32 |
| Bold | 0.17 | 0.23 | **0.71** | 0.21 |
| Effective | 0.27 | 0.26 | **0.60** | 0.30 |
| Direct/forceful/ gets own way | 0.37 | 0.32 | **0.51** | 0.28 |
| Independent | -0.05 | 0.40 | **0.47** | 0.22 |
| Insecure | 0.14 | -0.24 | **-0.61** | 0.19 |
| Cautious | 0.04 | -0.01 | **-0.65** | 0.26 |
| Timid | -0.12 | -0.14 | **-0.69** | 0.38 |
| Nervous/anxious/not calm | 0.21 | **0.40** | **-0.73** | 0.07 |
| Fearful | -0.01 | 0.05 | **-0.88** | 0.13 |
| Apprehensive | 0.08 | 0.15 | **-0.91** | 0.17 |
| Lazy | -0.20 | **-0.48** | -0.18 | **0.62** |
| Slow | -0.30 | -0.28 | -0.20 | **0.61** |
| Depressed | -0.17 | -0.14 | **-0.41** | **0.43** |

**S14 Table.** Long-tailed macaque factor structure for human situation ratings. Bolded items exceed a loading of |0.40|.

| | Factors | | | |
|---|---|---|---|---|
| **Items** | Apprehensive | Exploratory | Irritable | Lazy |
| Submissive/subordinate | **0.92** | 0.08 | -0.11 | -0.14 |
| Apprehensive | **0.89** | -0.02 | 0.08 | 0.15 |
| Fearful | **0.89** | -0.05 | 0.10 | 0.11 |
| Timid | **0.84** | -0.10 | -0.07 | 0.08 |
| Nervous/anxious/not calm | **0.79** | 0.19 | 0.40 | -0.13 |
| Cautious | **0.74** | -0.25 | -0.10 | 0.00 |
| Depressed | **0.64** | 0.02 | 0.22 | **0.51** |
| Insecure | **0.61** | -0.02 | -0.12 | -0.32 |
| Tense | **0.44** | -0.14 | 0.37 | 0.38 |
| Intelligent | **-0.41** | 0.34 | -0.08 | -0.32 |
| Strong | **-0.52** | 0.13 | 0.33 | 0.20 |
| Direct/forceful/gets own way | **-0.56** | **0.42** | 0.28 | 0.16 |
| Effective | **-0.59** | **0.42** | 0.18 | 0.09 |
| Bold | **-0.64** | 0.38 | 0.13 | -0.07 |
| Confident | **-0.75** | 0.31 | 0.07 | -0.01 |
| Sociable | -0.01 | **0.84** | -0.13 | -0.05 |
| Curious/exploratory/inquisitive | -0.16 | **0.78** | -0.01 | -0.17 |
| Active/Energetic | -0.08 | **0.72** | 0.08 | -0.27 |
| Affiliative/Companionable | -0.03 | **0.72** | -0.16 | -0.03 |
| Tolerant | -0.12 | **0.69** | **-0.55** | 0.17 |
| Opportunistic | -0.25 | **0.65** | 0.06 | -0.15 |
| Persistent | -0.33 | **0.62** | 0.18 | -0.01 |
| Defiant | -0.19 | **0.54** | **0.48** | 0.01 |
| Impulsive | 0.05 | **0.54** | **0.52** | -0.06 |
| Reckless | -0.10 | **0.52** | **0.46** | -0.07 |
| Solitary | 0.32 | **-0.46** | 0.05 | 0.35 |
| Irritable | 0.03 | -0.04 | **0.89** | 0.08 |
| Aggressive | -0.27 | 0.09 | **0.84** | 0.14 |
| Feisty | -0.22 | 0.02 | **0.84** | 0.05 |
| Excitable | 0.20 | 0.28 | **0.66** | -0.30 |
| Bullying | -0.38 | 0.31 | **0.58** | 0.12 |
| Unpredictable | 0.05 | 0.35 | **0.55** | -0.26 |
| Unemotional | **-0.44** | 0.01 | **-0.51** | 0.36 |
| Gentle | 0.27 | **0.47** | **-0.59** | 0.19 |
| Understanding | -0.24 | 0.10 | **-0.67** | -0.11 |
| Equable | -0.23 | 0.23 | **-0.69** | 0.32 |
| Calm/Equable | -0.25 | 0.18 | **-0.76** | 0.23 |
| Slow | -0.06 | -0.16 | -0.22 | **0.72** |
| Lazy | 0.27 | -0.33 | -0.09 | **0.61** |
| Independent | -0.29 | 0.18 | 0.32 | **0.50** |
| Vigilant | -0.13 | 0.37 | 0.18 | **-0.47** |

**S15 Table.** Bonnet macaque factor structure for human situation ratings. Bolded items exceed a loading of |0.40|.

| **Items** | **Factors** | | |
|---|---|---|---|
| | Effective | Lazy/ Exploratory | Apprehensive |
| Effective | **0.95** | 0.06 | 0.06 |
| Direct/forceful/ gets own way | **0.89** | 0.12 | -0.09 |
| Persistent | **0.84** | -0.10 | 0.11 |
| Defiant | **0.79** | -0.20 | 0.01 |
| Bullying | **0.78** | 0.15 | -0.04 |
| Reckless | **0.63** | -0.36 | 0.28 |
| Confident | **0.49** | -0.26 | -0.37 |
| Cautious | **-0.52** | 0.34 | 0.27 |
| Timid | **-0.68** | 0.20 | 0.23 |
| Submissive/subordinate | **-0.70** | -0.16 | 0.39 |
| Lazy | 0.04 | **0.89** | 0.04 |
| Slow | 0.11 | **0.88** | -0.02 |
| Depressed | -0.02 | **0.61** | 0.38 |
| Opportunistic | 0.28 | **-0.56** | 0.05 |
| Curious/exploratory/inquisitive | 0.03 | **-0.87** | -0.03 |
| Active/Energetic | 0.10 | **-0.87** | 0.08 |
| Nervous/anxious/not calm | 0.00 | -0.15 | **0.82** |
| Apprehensive | -0.13 | 0.30 | **0.67** |
| Tense | -0.31 | 0.22 | **0.55** |

**S16 Table.** Interfactor correlation coefficients (Phi) for the human situation factor models.

**Rhesus macaques**

| | Irritable$^{R}_{H}$ | Exploratory$^{R}_{H}$ | Apprehensive$^{R}_{H}$ |
|---|---|---|---|
| Exploratory$^{R}_{H}$ | 0.28 | – | – |
| Apprehensive$^{R}_{H}$ | 0.11 | 0.44 | – |
| Lazy$^{R}_{H}$ | -0.04 | 0.14 | 0.01 |

**Long-tailed macaques**

| | Apprehensive$^{L}_{H}$ | Exploratory$^{L}_{H}$ | Irritable$^{L}_{H}$ |
|---|---|---|---|
| Exploratory$^{R}_{H}$ | -0.55 | – | – |
| Irritable$^{L}_{H}$ | -0.01 | 0.17 | – |
| Lazy$^{L}_{H}$ | -0.01 | -0.20 | -0.16 |

**Bonnet macaques**

| | Effective$^{B}_{H}$ | Lazy/Exploratory$^{B}_{H}$ |
|---|---|---|
| Lazy/Exploratory$^{B}_{H}$ | -0.46 | – |
| Apprehensive$^{B}_{H}$ | -0.47 | 0.18 |

*S3 Text. Comparing Complex Item Human Situation Models, versus Simple Models*

Following our approach with the general models, we screened the human situation factor models to remove complex items following Howard [8]. We used Procrustes rotation prior to calculating congruence coefficients between the simple and complex models. For each of the species, factors from the simple models were highly congruent ($\Phi \geq 0.97$) with the original complex models (**S3 Fig**), with the exception of Exploratory$^{L}_{H}$, which still had *fair* congruence ($\Phi = 0.91$) with the simplified Exploratory$^{L}_{H}$ and *poor* congruence with Lazy$^{L}_{H}$ ($\Phi = -0.80$). These high congruence coefficients were despite a reduction of 12, 18, and 2 items in the simplified rhesus, long-tailed, and bonnet macaque models, respectively. The original number of factors were retained in the bonnet macaque models. In the simplified long-tailed macaque model, however, Exploratory$^{L}_{H}$ and Lazy$^{L}_{H}$ were merged.

**S17 Table.** Congruence between factors from the human situation models with complex items included or excluded. Simplified factors, from models with complex items are excluded, are italicized.

**Rhesus macaques**

| | Irritable$^{R}_{H}$ | Exploratory$^{R}_{H}$ | Apprehensive$^{R}_{H}$ | Lazy$^{R}_{H}$ |
|---|---|---|---|---|
| *Irritable$^{R}_{H}$* | 1.00 | 0.01 | 0.04 | -0.33 |
| *Exploratory$^{R}_{H}$* | 0.01 | 1.00 | 0.18 | 0.03 |
| *Apprehensive$^{R}_{H}$* | 0.04 | 0.19 | 1.00 | -0.08 |
| *Lazy$^{R}_{H}$* | -0.22 | 0.02 | -0.04 | 0.97 |

**Long-tailed macaques**

| | Apprehensive$^{L}_{H}$ | Exploratory$^{L}_{H}$ | Irritable$^{L}_{H}$ | Lazy$^{L}_{H}$ |
|---|---|---|---|---|
| *Apprehensive$^{L}_{H}$* | 0.97 | -0.40 | -0.02 | 0.01 |
| *Exploratory$^{L}_{H}$* | -0.40 | 0.91 | 0.07 | -0.80 |
| *Irritable$^{L}_{H}$* | -0.02 | 0.07 | 1.00 | 0.04 |

**Bonnet macaques**

| | Effective$^{B}_{H}$ | Lazy/ Exploratory$^{B}_{H}$ | Apprehensive$^{B}_{H}$ |
|---|---|---|---|
| *Effective$^{B}_{H}$* | 1.00 | -0.11 | -0.16 |
| *Lazy/ Exploratory$^{B}_{H}$* | -0.11 | 1.00 | 0.10 |
| *Apprehensive$^{B}_{H}$* | -0.16 | 0.10 | 1.00 |

*S4 Text. Influence of Stepwise Exclusion of Items on Congruence, Human Situation Models*

We sought to determine whether particular items were contributing to incongruence for items with similar item structure. We first re-obtained congruence between the factors after removing a single item. We used these leave-one-out congruence estimates to determine which item removal most greatly improved congruence. We then repeated this process of item exclusion until we attained at least *poor* congruence (≥0.80).

For $\text{Lazy}^{RL}{}_{H}$ congruence was improved by removal of: insecure. We were able to improve to *fair* congruence with the additional removal of: cautious, understanding, and excitable, in descending order of the contribution to incongruence.

**S18 Table.** Items with scores < |0.40| in our fuzzy set analyses for four factors in our human situation factor models. See **Table 4** for the remaining items.

| | Factors | | | |
|---|---|---|---|---|
| **Items** | Exploratory | Apprehensive | Lazy | Irritable* |
| Cautious | -0.01 | 0.27 | 0.00 | 0.04 |
| Confident | 0.24 | -0.37 | -0.01 | 0.00 |
| Direct/forceful/ gets own way | -0.12 | -0.09 | 0.12 | 0.28 |
| Effective | -0.06 | 0.06 | 0.06 | 0.18 |
| Independent | 0.18 | -0.29 | 0.22 | -0.05 |
| Intelligent | 0.34 | -0.23 | -0.05 | -0.08 |
| Persistent | 0.10 | 0.11 | -0.01 | 0.18 |
| Submissive/subordinate | 0.08 | 0.39 | -0.14 | -0.11 |
| Timid | -0.10 | 0.23 | 0.08 | -0.07 |
| Unpredictable | 0.35 | -0.02 | -0.04 | 0.37 |
| Vigilant | 0.37 | -0.13 | -0.01 | 0.05 |

*These items were only present in rhesus and long-tailed macaques.

**S19 Table.** Comparison of our rhesus macaque general personality factor structure, relative to published results using a similar, albeit reduced, survey.

| | This Study | Capitanio et al. (1999) | Maninger et al. (2003) | Capitanio et al. (2005) |
|---|---|---|---|---|
| **Subjects (N by Sex)** | 48 M; 106 F | 42 M | 88 M | 58 M |
| **Survey Item Count** | 51 | 25 | 50 | 50 |
| Aggressive | Irritable (+) | Confident (+) | - | - |
| Confident | Irritable (+) | Confident (+) | Confidence (+) | Confident (+) |
| Excitable | Irritable (+) | Excitable (+) | - | - |
| Irritable | Irritable (+) | - | Irritability (+) | Irritability (+) |
| Bold | Confident (-) / Irritable (+) | - | Confidence (+) | Confident (+) |
| Cautious | Confident (+) | - | Confidence (-) | - |
| Direct/ Forceful/ Gets own way | Confident (-) / Irritable (+) | - | Confidence (+) | Confident (+) |
| Effective | Confident (-) | Confident (+) | - | - |
| Fearful | Confident (+) | - | Confidence (-) | - |
| Submissive/ Subordinate | Confident (+) | Excitable (+) / Confident (-) / Equable (+) | Confidence (-) | Confident (+) |
| Timid | Confident (+) | | Confidence (-) | Confident (+) |
| Affiliative/ Companionable | Sociable (+) | - | Sociable (+) | Sociability (+) |
| Sociable | Sociable (+) | Sociable (+) | - | - |
| Solitary | Sociable (-) / Equable (+) | - | Sociable (-) | Sociability (+) |
| Warm/ Affectionate | Sociable (+) | - | Sociable (+) | Sociability (+) |
| Active/ Energetic | Active (-) | Excitable (+) | - | - |
| Curious/ Exploratory/ Inquisitive | Active (-) / Equable (+) | Sociable (+) / Confident (+) | - | - |
| Slow | Active (-) | Equable (+) / Excitable (-) | Equability (+) | Equability (+) |
| Calm/ Equable | Equable (+) | - | Equability (+) | Equability (+) |
| Equable | Equable (+) | Equable (+) | - | - |
| Playful | Equable (+) / Active (-) | Sociable (+) | - | - |
| Understanding | Equable (+) | Equable (+) | - | - |
| * Tense | - | - | Confidence (-) | - |
| * Reckless | - | - | Irritability (+) | Irritability (+) |

* these survey items were not included in our factor models, but were present in prior work

## Supplementary References

1. McCrae RR, Weiss A. Observer Ratings of Personality. In: Robins RW, Farley RC, Krueger RF, editors. Handbook of research methods in personality psychology. New York, NY, USA: The Guilford Press; 2007. p. 259–72.

2. LeBreton JM, Senter JL. Answers to 20 Questions About Interrater Reliability and Interrater Agreement. Organizational Research Methods. 2008;11(4):815–52.

3. Tinsley HE, Weiss DJ. Interrater reliability and agreement. In: Handbook of applied multivariate statistics and mathematical modeling [Internet]. Elsevier; 2000 [cited 2024 May 9]. p. 95–124. Available from: https://www.sciencedirect.com/science/article/pii/B9780126913606500057

4. McGraw KO, Wong SP. Forming inferences about some intraclass correlation coefficients. Psychological methods. 1996;1(1):30–46.

5. Eckardt W, Steklis HD, Steklis NG, Fletcher AW, Stoinski TS, Weiss A. Personality dimensions and their behavioral correlates in wild Virunga mountain gorillas (*Gorilla beringei beringei*). Journal of Comparative Psychology. 2015;129(1):26–41.

6. Koski SE. Broader horizons for animal personality research. Front Ecol Evol. 2014;2:70.

7. Weiss A, Wilson ML, Collins DA, Mjungu D, Kamenya S, Foerster S, et al. Personality in the chimpanzees of Gombe National Park. Sci Data. 2017 Oct 24;4(1):170146.

8. Howard MC. A review of exploratory factor analysis decisions and overview of current practices: What we are doing and how can we improve? International Journal of Human-Computer Interaction. 2016;32(1):51–62.
